# Antimicrobial, antioxidant and cytotoxic evaluation of diazenyl chalcones along with insights to mechanism of interaction by molecular docking studies

**DOI:** 10.1186/s13065-019-0596-5

**Published:** 2019-07-09

**Authors:** Harmeet Kaur, Jasbir Singh, Balasubramanian Narasimhan

**Affiliations:** 1 Faculty of Pharmaceutical Sciences, Maharshi Dayanand University, Rohtak, 124001 India; 2 College of Pharmacy, Postgraduate Institute of Medical Sciences, Rohtak, 124001 India

**Keywords:** Chalcones, Antimicrobial, Diazenyl, Antioxidant, Cytotoxicity

## Abstract

**Background:**

In continuation of our work, new diazenyl chalcones scaffolds (**C-18** to **C-27**) were efficiently synthesized from substituted acetophenone azo dyes (**A**–**E**) by base catalyzed Claisen–Schmidt condensation with different substituted aromatic/heteroaromatic aldehydes.

**Methodology:**

The synthesized chalcones were assessed for their in vitro antimicrobial potential towards several pathogenic microbial strains by tube dilution method and further evaluated for antioxidant potential by DPPH assay. These derivatives were also assessed for the cytotoxicity towards the human lung cancer cell line (A549) and normal cell line (HEK) by MTT assay. The most active antimicrobial compounds were docked using Schrodinger *v*18.1 software with the various potential bacterial receptors to explore the mechanism of interaction.

**Results:**

The derivative **C-22** exhibited high antibacterial activity with very low MIC (1.95–3.90 µg ml^−1^) and MBC (3.90–7.81 µg ml^−1^) values. The derivatives **C-23**, **C-24** and **C-27** have demonstrated good antioxidant potential (IC_50_ = 7–18 µg ml^−1^) correlated to the ascorbic acid (IC_50_ = 4.45 µg ml^−1^). The derivative **C-25** had shown comparable cytotoxicity to camptothecin against A549 cell line. The docking studies predicted the bacterial dihydrofolate reductase (PDB ID: 3SRW) and bacterial DNA gyrase (PDB ID: 4ZVI) as the possible targets for most of the active antimicrobial compounds. These derivatives affirmed their safety by presenting less cytotoxicity towards HEK cells. Further the ADME prediction by qikprop module of the Schrodinger proved that these compounds exhibited drug-like attributes.

**Conclusion:**

Hence, these compounds have shown their potential as lead for future expansion of novel antimicrobial and cytotoxic drugs.
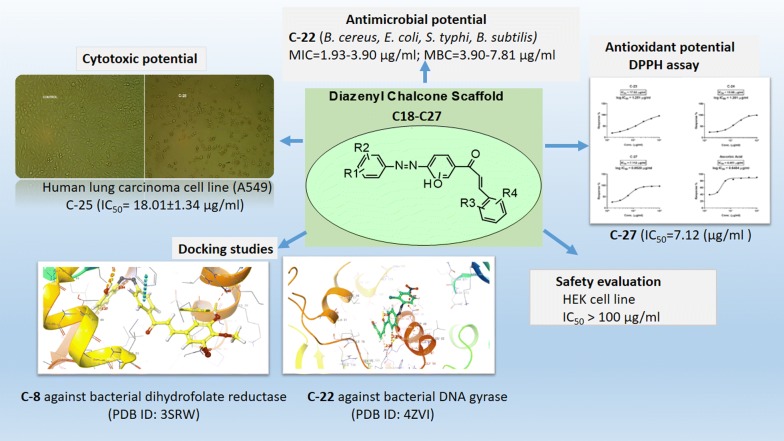

**Electronic supplementary material:**

The online version of this article (10.1186/s13065-019-0596-5) contains supplementary material, which is available to authorized users.

## Introduction

The use of antimicrobial agents in clinical practice is one of the remarkable achievements of modern medicine. Several new antimicrobials became available to treat many infectious diseases in the last half of the 20th century. Also, due to the wide range of these antimicrobials, the clinicians are provided with a wide scope of choices to treat different infectious diseases [1, 2]. It is significant from the drop in death rate, i.e. from 797 per lakh to 36 per lakh humans, a decrease by a factor of greater than 20 during the period spanning from 1900 to 1980. It is a clear cut proof of the efficacy and testimony of these antibiotics. However, the microorganisms have developed resistance due to the irrational use of antimicrobials over a period of time. This is evident from the rise of drug-resistant microbial pathogens like methicillin-resistant *Staphylococcus aureus* (MRSA), vancomycin resistant enterococci, and multi-drug resistant tuberculosis [3–5]. Microbial resistance has been emerged as the most pressing health issue and has become a significant challenge as far as the delivery of healthcare is concerned. The emergence of new strains of microorganisms is also contributing to the observed resistance to the antimicrobial compounds [6, 7]. This spread of resistance has thus restricted the treatment alternatives for some severe and life-threatening infectious disorders [8, 9]. Therefore, in parallel to the discovery and expansion of new antimicrobial drugs, there is also a requirement for the proper use of current antimicrobials in consideration to decrease the spread of antimicrobial resistance. As there is an urgency for new antimicrobial agents, the current scenario is that many pharmaceutical companies are opting out of the antimicrobial section, especially antibacterial drug development [10]. The present situation has been further worsened as other malignant diseases such as cancer in which the host has immune-compromised or concomitant illness frequently accompanied by the microbial infections [11, 12]. The chances of infections in cancer patients are higher in comparison to others due to interrupted epithelial barriers, decrease in neutrophils, compromised host defence, and changes in the microbial flora, etc. [13, 14]. Among cancers, lung cancer is the utmost prevalent form and the most common cause of death in men and women worldwide. Non-small cell lung cancer (NSCLC) cells are inherently resistant to some commonly used cytotoxic drugs; however, small-cell lung cancer (SCLC) cells can develop resistance on continuous use of the drugs. Most of the patients with lung cancer have already developed metastatic disease, at the time of diagnosis, hence limiting the use of another therapeutic option, such as radiation and surgery. The current drugs used for the treatment of lung cancers have also developed resistance, hence conferred the limited treatment scope [15, 16]. All the above facts necessitate the need to understand the basic mechanism of drug interaction with the possible targets on the molecular level for the development of new agents having antimicrobial and cytotoxic potential.

Chalcone derivatives, the main class of compounds, have gained immense interest from bioorganic and medicinal chemistry research. The chalcones are characterized by three carbon α, β-unsaturated carbonyl system joined by two aromatic rings. Chalcones also constitute an important class of natural products having considerable pharmacological potential [17, 18]. The various biological activities like antimicrobial, antibacterial, antifungal, anti-inflammatory, antimalarial, antileishmanial, antioxidant and antitubercular etc. have been reported for the compounds comprising of chalcone backbone. The antimicrobial property of the chalcones is generally correlated with the reactive α, β-unsaturated keto function in the molecule [19, 20].

Azo dyes are the most widely used class of colouring materials owing to various applications in various fields such as dying textile fibres, the colouring of different materials, biomedical studies and in advanced organic synthesis [21, 22]. Azo compounds and their derivatives are also known for their use as antibacterial, antifungal, antidiabetics, antineoplastics, anti-inflammatory, antiseptic and other useful chemotherapeutic agents [23, 24]. Several azo compounds particularly synthesized from β-naphthol, *m*-resorcinol, tyrosine, aspirin, paracetamol etc. have been frequently reported and exhibited impressive biocidal effects [25, 26].

In the light of above facts, herein, we further extend our synthesis to some novel diazenyl chalcones and evaluation of their antimicrobial, antioxidant and cytotoxic potential against lung cancer cell line (A549) and safety study against normal cell line (HEK 293). To further explore their antimicrobial mechanism, we dock the active antimicrobial compounds from this series and the already synthesized compounds by Kaur and Narasimhan [27] against several potential receptors in bacteria.

## Results and discussion

### Chemistry

**Scheme 1:**
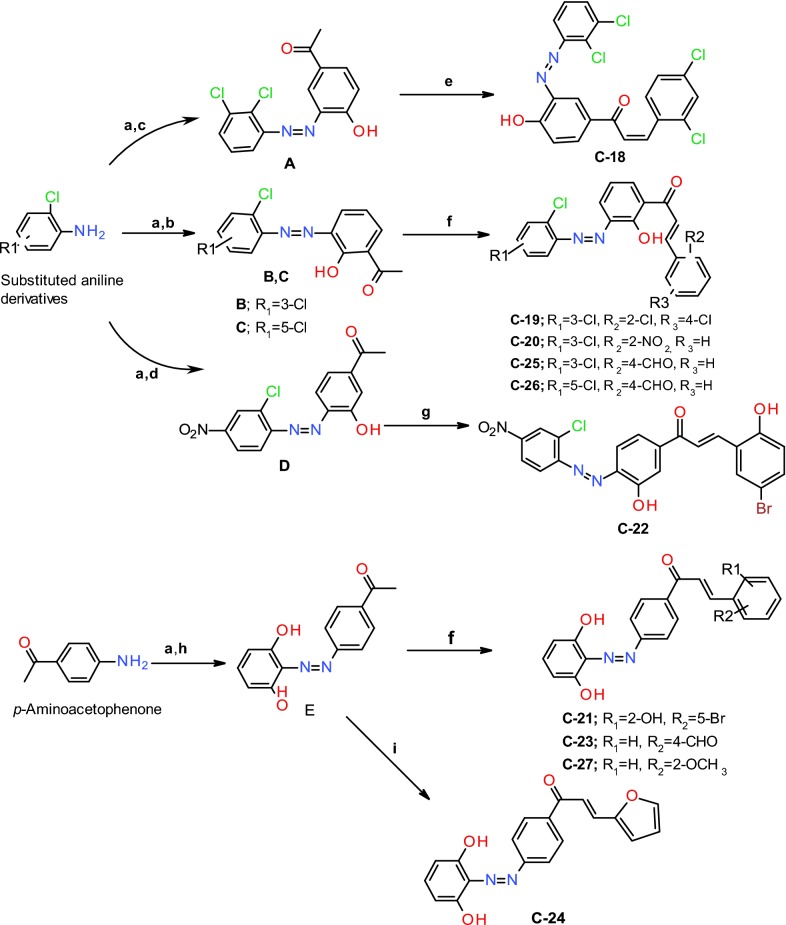
Synthesis of diazenyl chalcones (**C18**–**C-27**). Reaction and reagents: (a) sodium nitrite, HCl, 0 °C, (b) o-hydroxyacetophenone, ethanol, 10% NaOH; (c) p-hydroxyacetophenone, ethanol, 10% NaOH; (d) m-hydroxyacetophenone, ethanol, 10% NaOH; (e) 2,4 dichlorobenzaldehyde, ethanol, 7–8 drops of 50% NaOH, stirring for 16 h at rt; (f) different mono or di-substituted benzaldehydes, ethanol, 7–8 drops of 50% NaOH, stirring for 18–24 h at rt; (g) 5-bromosalicylaldehyde, ethanol, 7–8 drops of 50% NaOH, stirring for 18 h at rt; (h) resorcinol, ethanol, 10% NaOH; (i) furfural, ethanol, 7–8 drops of 50% NaOH, stirring for 20 h at rt

The synthesis of diazenyl chalcones (**C18**–**C27**) has been presented in Scheme 1. The anilines with mono or di-substitution (generally with chloro and nitro groups) were diazotized in the presence of HCl and NaNO_2_, then coupled with *o*, *p* and *m* hydroxy substituted acetophenone derivative in the presence of ethanolic alkaline solution to give azo dyes (**A**–**D**). The azo dye **E** was synthesized by diazotization of *p*-aminoacetophenone followed by coupling with resorcinol in ethanolic solution. The diazenyl chalcones (**C18**–**C27** were synthesized by the Claisen–Schmidt condensation of the α-methyl group (adjacent to keto group) present in azo dye with aldehyde group present in various reactants in the presence of alkali solution (Scheme 1). The target derivatives structures were confirmed by UV–Vis, FTIR, mass spectroscopy, NMR spectroscopy, and elemental analysis.

### Characterization by spectroscopy

The FTIR spectra of dyes (**A**–**E**) and diazenyl chalcones (**C18**–**C27**) were recorded by the KBr pellet method. The aliphatic C-H stretch in dyes and diazenyl chalcones were observed at 3100–3035 cm^−1^. The –C=C– stretching vibration was assigned to bands at 1615–1565 cm^−1^. A broad peak was noticed for the phenolic group at 3450–3150 cm^−1^. The carbonyl stretching vibrations for the enones was found between 1640 and 1685 cm^−1^. The C-O stretching vibration was assigned in the range of 1300–1340 cm^−1^. The –N=N– linkage was observed at 1400–1471 cm^−1^. Another peaks noticed were Ar–O stretching at 1120–1290 cm^−1^, –C=C– bending vibration at 670–780 cm^−1^, the C–N stretching at 1050–1360 cm^−1^. Two strong bands at 1520–1530 cm^−1^ and 1335–1350 cm^−1^ attested the existence of NO_2_ stretch. The band observed at 1050–550 cm^−1^ affirmed the absorption of C–X (halogen). The NMR spectra (^1^H NMR and ^13^C NMR) of the derivatives (**C18**–**C27**) were recorded in deuterated DMSO. The protons of the double bond of the enone moiety in most of the derivatives appeared as doublets or multiplet in the range of 5.0–6.21 ppm and 5.67–6.72 ppm. A broad peak in the range of 10–14 Hz was observed for the proton of the phenolic group. The protons of the –OCH_3_ group were found as a singlet in the range of 3.6 ppm. The other aromatic ring protons appeared in the range of 6.50–8.52 ppm. The carbon signals of carbonyl (C=O) carbon in different diazenyl chalcones came in the range of 202–190 ppm. The signal of the hydroxy substituted carbon atom was noticed at 156–165 ppm. The carbon signals of other aromatic carbons were observed in the range of 135–110 ppm depending on the nature of substituents. The methoxy and methyl carbons were detected at 56 ppm and 23–27 ppm, respectively. The proton and carbon spectra of selected compounds have been provided in Additional file 1. The mass spectroscopy was done to confirm the molecular mass of the synthesized compounds. The % of carbon, oxygen nitrogen and hydrogen in the diazenyl chalcones was detected within the marked limits.

### Antimicrobial assay

The MIC and MBC/MFC values were determined for the synthesized diazenyl chalcones (**C18**–**C27**) by serial tube dilution method in comparison with controls (dye A and dye E and chalcone ((*E*)-3-(2,4-dichlorophenyl)-1-(4-hydroxyphenyl)prop-2-en-1-one)) and various standard drugs, i.e., cefadroxil, cefotaxime, ciprofloxacin (CIP), trimethoprim/sulfamethoxazole (SXT) and fluconazole. The results have been presented in µg ml^−1^ in Tables 1 and 2, respectively. The results declared that the diazenyl chalcone **C-22** had shown the highest activity towards a maximum number of tested bacterial strains in comparison to the controls and standard drugs with MIC ranges from the 1.95–3.90 µg ml^−1^ and MBC ranges from 3.90 to 7.81 µg ml^−1^. Hence, the derivative **C-22** acted as the bacteriostatic agent but also as a bactericidal agent. The derivatives **C-18**, **C-21**, **C-23**, **C-24** and **C-27** had also shown higher activity towards *E. coli* with MIC ranges from 7.81 to 15.62 µg ml^−1^ in comparison to the controls. The derivative **C-24** had also exhibited activity towards *B. cereus, S. typhi* and *S. aureus* with MIC ranges from 7.81 to 15.62 µg ml^−1^, and MBC ranges from 15.62 to 31.25 µg ml^−1^. The derivatives **C-19** and **C-27** had also shown bacteriostatic action towards *B. subtilis*, with MIC of 15.62 µg ml^−1^. The synthesized chalcones had shown moderate activity (IC_50_ = 31.25–125 µg ml^−1^) against fungal strains in comparison with fluconazole (IC_50_ = 7.81–15.62 µg ml^−1^). The other synthesized derivatives showed moderate to low activity against different tested microbial strains. The derivative **C-22** having substituted nitro, chloro and bromo groups exhibited the maximum antimicrobial activity.

**Table 1:** MIC (µg ml^−1^) of diazenyl chalcones (**C18**–**C27**) against various microbial strains

Compound	*B. cereus* MIC (µg ml^−1^)	*S. typhi* MIC (µg ml^−1^)	*E. coli* MIC (µg ml^−1^)	*S. aureus* MIC (µg ml^−1^)	*B. subtilis* MIC (µg ml^−1^)	*C. albicans* MIC (µg ml^−1^)	*A. fumigatus* MIC (µg ml^−1^)
**C-18**	31.25	31.25	15.62	31.25	125	125	125
**C-19**	500	125	500	125	15.62	125	125
**C-20**	125	125	125	125	62.5	125	125
**C-21**	62.5	31.25	15.62	125	31.25	62.5	125
**C-22**	3.90	3.90	1.95	3.90	1.95	31.25	62.5
**C-23**	62.5	31.25	15.62	125	62.5	125	62.5
**C-24**	15.62	7.58	15.62	15.62	31.25	62.5	125
**C-25**	125	125	62.5	62.5	15.62	31.25	62.5
**C-26**	250	15.62	62.5	7.81	62.5	125	125
**C-27**	125	62.5	7.81	62.5	15.62	62.5	125
**Dye A**	31.25	62.5	15.62	125	125	62.5	125
**Dye E**	62.5	125	31.25	31.25	62.5	125	125
Chalcone	62.5	31.25	15.62	31.25	62.5	125	125
Cefadroxil	31.25	125	31.25	15.62	62.5	–	–
Cefotaxime	7.81	3.90	7.81	15.62	1.95	–	–
SXT	3.90	3.90	1.95	7.81	7.81	–	–
CIP	7.81	7.81	3.90	1.95	7.81	–	–
Fluconazole	–	–	–	–	–	7.81	15.62

*Chalcone* (*E*)-3-(2,4-dichlorophenyl)-1-(4-hydroxyphenyl)prop-2-en-1-one,* SXT* Sulfamethoxazole/trimethoprim; CIP, ciprofloxacin

**Table 2:** MBC/MFC (µg ml^−1^) of diazenyl chalcones (**C18**–**C27**) against various microbial strains

Compound	*B. cereus* MBC (µg ml^−1^)	*S. typhi* MBC (µg ml^−1^)	*E. coli* MBC (µg ml^−1^)	*S. aureus* MBC (µg ml^−1^)	*B. subtilis* MBC (µg ml^−1^)	*C. albicans* MFC (µg ml^−1^)	*A. fumigatus* MFC (µg ml^−1^)
**C-18**	125	125	62.5	125	125	125	125
**C-19**	500	125	500	125	62.5	125	250
**C-20**	125	125	250	125	125	250	125
**C-21**	62.5	125	31.25	125	62.5	125	125
**C-22**	3.90	7.81	7.81	31.25	7.81	31.25	125
**C-23**	62.5	62.5	15.62	125	62.5	125	125
**C-24**	15.62	15.62	31.25	15.62	62.5	125	125
**C-25**	125	125	125	62.5	62.5	62.5	62.5
**C-26**	250	62.5	125	31.25	62.5	125	125
**C-27**	125	62.5	15.62	62.5	31.25	62.5	125
Dye A	31.25	62.5	31.25	125	125	62.5	125
Dye E	62.5	125	31.25	62.5	125	125	125
Chalcone	62.5	62.5	31.25	62.5	62.5	125	125
Cefadroxil	31.25	125	31.25	31.25	125	–	–
Cefotaxime	15.62	7.81	7.81	31.25	7.81	–	–
SXT	7.81	3.90	1.95	7.81	15.62	–	–
CIP	15.62	7.81	7.81	3.90	15.62	–	–
Fluconazole	–	–	–	–	–	31.25	125

*Chalcone* (*E*)-3-(2,4-dichlorophenyl)-1-(4-hydroxyphenyl)prop-2-en-1-one,* SXT* Sulfamethoxazole/trimethoprim; *CIP* ciprofloxacin

### Antioxidant evaluation

**Fig. 1:**
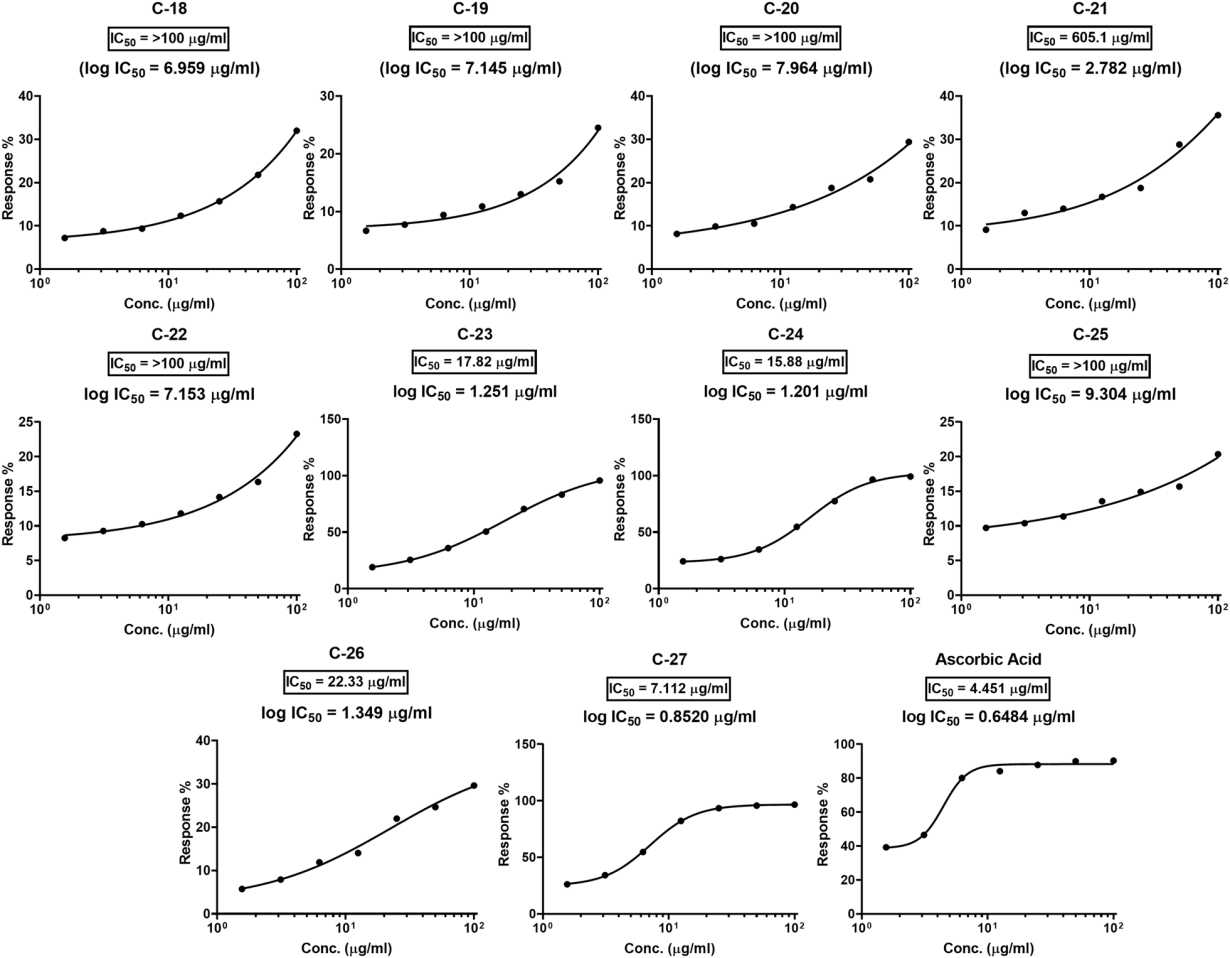
Antioxidant activity of different diazenyl chalcones as function of concentration in DPPH assay

DPPH scavenging activity results displayed that title compounds showed very low to high antioxidant activity and presented in Table 3. The % inhibition of DPPH was plotted against the logarithmic values of the concentration of test samples and the standard, to find the IC_50_ (µg ml^−1^), which is the quantity of compound required to inhibit the absorbance of free radical DPPH by 50%. These graphs are presented in Fig. 1. Among all, the derivative **C-27** had shown the highest antioxidant activity with IC_50_ of 7.12 µg ml^−1^ comparable to the ascorbic acid (IC_50_ = 4.45 µg ml^−1^). The other derivatives **C-23** and **C-24** had also shown the good antioxidant activity with the IC_50_ of 15–18 µg ml^−1^ as compared to the controls (dyes and chalcone derivative). The most of other tested derivatives exhibited less antioxidant activity by presenting IC_50_ > 100 µg ml^−1^. The DPPH assay results indicated that the compounds with the substituted resorcinol ring exhibited the highest antioxidant activity.

**Table 3:** % inhibition of DPPH by diazenyl chalcones (**C18**–**C27**)

Compound	100 (µg ml^−1^) % inhibition	50 (µg ml^−1^) % inhibition	25 (µg ml^−1^) % inhibition	12.5 (µg ml^−1^) % inhibition	6.25 (µg ml^−1^) % inhibition	3.12 (µg ml^−1^) % inhibition	1.56 (µg ml^−1^) % inhibition	IC_50_ (µg ml^−1^) % inhibition
**C-18**	31.98	21.79	15.68	12.35	9.37	8.76	7.21	> 100
**C-19**	24.49	15.22	12.99	10.87	9.39	7.71	6.64	> 100
**C-20**	29.39	20.76	18.96	14.32	10.51	9.87	8.15	> 100
**C-21**	39.56	35.78	30.75	25.71	20.98	12.99	8.71	> 100
**C-22**	23.27	16.34	14.15	11.79	10.25	9.26	8.25	> 100
**C-23**	95.68	83.17	70.53	50.45	35.86	25.53	19.03	17.82
**C-24**	99.17	96.46	77.38	54.71	34.65	26.13	24.16	15.88
**C-25**	20.34	15.67	14.91	13.56	11.36	10.38	9.71	> 100
**C-26**	29.62	24.65	21.98	14.04	11.90	7.91	5.72	> 100
**C-27**	96.62	95.71	93.45	82.21	54.67	34.18	26.13	7.12
Dye A	35.45	29.87	22.18	17.67	11.97	9.23	4.65	> 100
Dye E	56.78	47.56	34.89	26.18	19.80	12.68	7.89	69.40
Chalcone	51.80	34.56	21.57	16.90	10.76	8.12	6.54	76.77
Ascorbic acid	90.31	89.95	87.81	84.09	80.07	46.53	39.22	4.45

*Chalcone* (*E*)-3-(2,4-dichlorophenyl)-1-(4-hydroxyphenyl)prop-2-en-1-one

### Cytotoxicity results

**Fig. 2:**
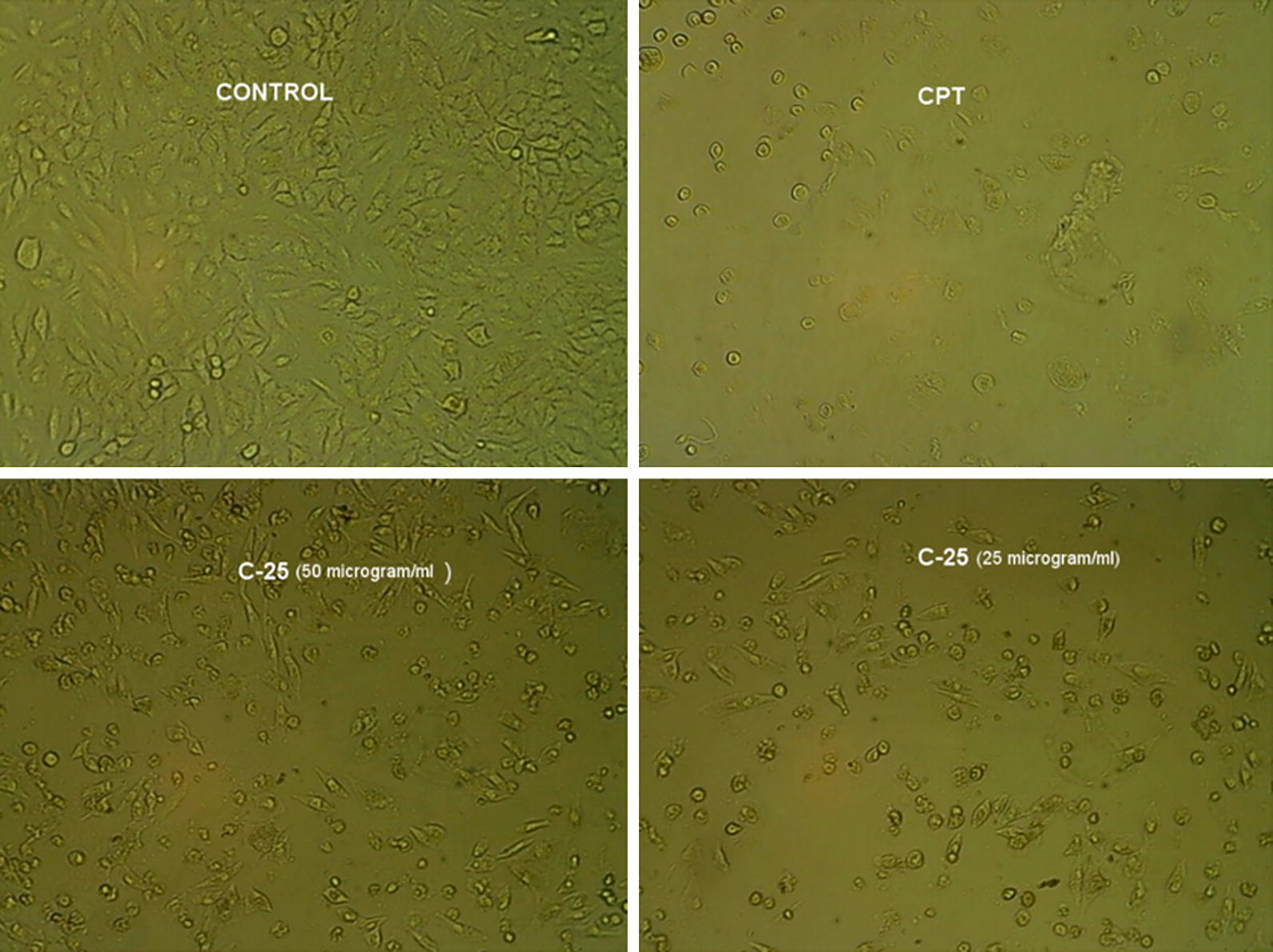
Morphological changes in A549 cell line on treatment with Camptothecin (CPT), **C-25** at 50 µg ml^−1^ and **C-25** at 25 µg ml^−1^

The cytotoxicity of the synthesized diazenyl chalcone derivatives (**C-19**, **C-22**, **C-23**, **C-24**, **C-25** and **C-27**) was evaluated against A-549 cell line by MTT assay using reference drug camptothecin (CPT). The IC_50_ values were calculated from the cell viability graphs and presented in Table 4. The cytotoxicity results revealed that the derivative **C-25** exhibited good cytotoxic potential having IC_50_ of 18.01 µg ml^−1^ towards A549 cell line in comparison to the standard drug (IC_50_ = 8.7 µg ml^−1^). Figure 2 indicated that the diazenyl chalcone **C-25** had reduced the number and clumping of A549 cells to a significant extent. The other tested derivatives were found to be less active against tested lung cancer cell line with IC_50_ > 100 µg ml^−1^. The compounds were also evaluated for the possible cytotoxicity against normal cell line (HEK) by MTT assay. The tested compounds revealed their safety by exhibiting low cytotoxicity towards HEK cell line with IC_50_ > 100 µg ml^−1^. The test derivative **C-25** presented the higher selectivity index by exhibiting IC_50_ ratio of normal cell line (HEK293) to carcinoma cell line 15 times as compared to the camptothecin.

**Table 4:** IC_50_ (µg ml^−1^) of synthesized diazenyl chalcones against A549 and HEK cell line

Compound	A549 IC_50_ (µg ml^−1^)	HEK IC_50_ (µg ml^−1^)	Ratio (HEK/A549) IC_50_ (µg ml^−1^)
**C-19**	140.17 ± 1.11	221.06 ± 3.23	1.58
**C-22**	156.45 ± 1.23	296.45 ± 2.32	1.90
**C-23**	171.31 ± 2.46	190.11 ± 1.56	1.11
**C-24**	108.89 ± 3.21	280.45 ± 1.21	2.59
**C-25**	18.01 ± 1.34	270.23 ± 2.82	15.00
**C-27**	123.52 ± 2.56	330.56 ± 2.90	2.67
Std	8.7	9.0 [28]	1.03

*Std* camptothecin,* A549* human lung cancer cell line,* HEK* human embryonic kidney cells

### Molecular docking results

**Fig. 3:**
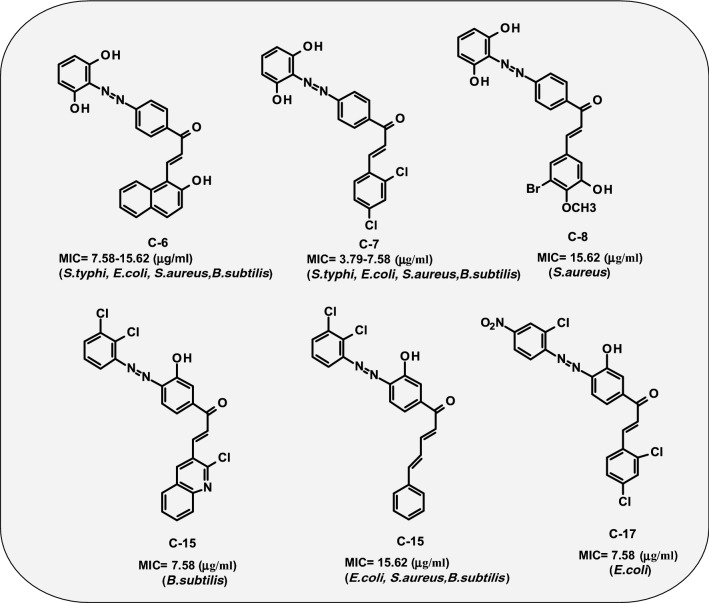
Structures of more active antimicrobial derivatives synthesized by Kaur et al. [27]

With the view to elucidate the mode of interaction of active compounds with the bacterial targets, the active compounds from this series and from already synthesized series (structures presented in Fig. 3) [27] were docked on the various essential bacterial proteins such as DNA topoisomerase from *E. coli* (PDB ID: 3FV5), dihydropteroate synthase from *S. pneumonia* (PDB ID: 2VEG) [29], dihydrofolate reductase from *S. aureus* (PDB ID: 3SRW) [30], DNA gyrase B from *E. coli* (PDB ID: 4ZVI) [31], UDP-*N*-acetylmuramoyl-l-alanine:d-glutamate ligase (PDB ID: 1UAG) [32]. The GLIDE module was used to carry out the molecular docking study, and the findings were examined based on glide energy, and docking score and presented in Tables 5 and 6 respectively. The obtained docking poses were examined visually, and the interactions of the molecules with the residues of the binding pocket were studied with the help of ligand-interactions diagrams. The docking scores have been presented in terms of negative values, lower the docking score and glide energy, best would be the binding affinity. The docking scores of the test derivatives and standard drugs were analyzed for the various bacterial protein targets, and the majority of the active compounds showed high docking scores against dihydrofolate reductase enzyme in comparison to trimethoprim. The highest docking score and binding energy was shown by the derivative **C-8** (− 9.221, − 51.794 kcal mol^−1^) followed by **C-6** (− 8.91, − 53.318 kcal mol^−1^) and **C-22** (− 7.544, − 62.888 kcal mol^−1^) as compared to trimethoprim (− 8.10, − 36.186 kcal mol^−1^). The most active compound **C-22** from this series had shown less interaction with the most of the other proteins but had shown highest docking score (− 4.174, − 44.539 kcal mol^−1^) against bacterial DNA gyrase B subunit as compared to the standard drug ciprofloxacin (− 3.284, − 48.864 kcal mol^−1^). The derivative **C-21** exhibited high docking score (− 7.095, − 48.855 kcal mol^−1^) against bacterial cell wall proteins followed by **C-6** (− 6.816, − 52.199 kcal mol^−1^) in comparison to methicillin (− 4.07, − 40.194 kcal mol^−1^).

**Table 5:** Glide energy and docking scores of active diazenyl chalcones against bacterial targets

Compd	Dihydrofolate reductase (PDB ID: 3SRW)		Bacterial DNA gyrase (PDB ID: 4ZVI)		UDP-*N*-Acetylmuramoyl-l-alanine:D-glutamate ligase (PDB ID: 1UAG)	
	Glide energy	Docking score	Glide energy	Docking score	Glide energy	Docking score
**C-6**	− 53.318	− 8.905	− 40.196	− 2.998	− 52.199	− 6.816
**C-7**	− 48.033	− 6.733	− 48.864	− 3.284	− 44.529	− 5.43
**C-8**	− 51.794	− 9.221	− 40.172	− 2.785	− 47.141	− 4.578
**C-15**	− 51.925	− 7.884	− 36.247	− 2.811	− 52.048	− 6.752
**C-16**	− 46.398	− 6.641	− 42.473	− 3.998	− 48.58	− 6.623
**C-17**	− 53.159	− 5.404	− 48.849	− 2.952	− 54.593	− 5.738
**C-20**	− 44.995	− 7.949	− 37.737	− 2.292	− 47.809	− 5.426
**C-21**	− 48.438	− 7.822	− 39.35	− 0.388	− 48.855	− 7.095
**C-22**	− 62.888	− 7.544	− 44.539	− 4.174	− 47.117	− 2.358
**C-23**	− 51.842	− 7.466	− 38.982	− 3.514	− 43.714	− 5.357
**C-24**	− 49.104	− 8.713	− 37.46	− 3.745	− 45.616	− 5.746
**C-25**	− 52.339	− 8.762	− 34.594	− 1.489	− 40.456	− 4.682
**C-27**	− 55.625	− 8.632	− 44.69	− 3.645	− 51.016	− 6.063
Std 1	− 48.417	− 5.255	− 36.5	− 4.146	− 46.549	− 5.557
Std 2	− 53.011	− 5.387	− 46.046	− 4.137	− 57.925	− 5.537
Std 3	− 45.97	− 7.349	− 48.864	− 3.284	− 36.076	− 5.147
Std 4	− 36.186	− 8.106	− 36.281	− 4.086	− 33.133	− 5.702
Std 5	–	–	–	–	− 40.194	− 4.07

*Std 1* cefadroxil,* Std 2* cefotaxime,* Std 3* ciprofloxacin,* Std 4* trimethoprim,* Std 5* methicillin

**Table 6:** Glide energy and docking scores of active diazenyl chalcones against bacterial targets

Compound	Topoisomerase IV (PDB ID: 3FV5)		Dihydropteroate synthase (PDB ID: 2VEG)	
	Glide energy	Docking score	Glide energy	Docking score
**C-6**	− 35.998	− 4.31	− 43.185	− 3.731
**C-7**	− 39.459	− 4.521	NA	NA
**C-8**	− 38.57	− 4.738	− 38.526	− 3.367
**C-15**	− 39.441	− 3.873	− 41.302	− 3.072
**C-16**	− 37.987	− 2.875	− 37.295	− 3.502
**C-17**	− 44.891	− 2.783	− 37.847	− 1.543
**C-20**	− 32.631	− 2.788	− 38.144	− 4.52
**C-21**	− 39.464	− 3.94	− 38.013	− 4.164
**C-22**	− 41.831	− 2.544	− 38.886	− 2.984
**C-23**	− 38.67	− 2.878	− 36.966	− 3.293
**C-24**	− 37.802	− 3.391	− 39.174	− 3.44
**C-25**	− 35.409	− 3.073	− 38.97	− 3.566
**C-26**	− 44.192	− 2.48	− 43.524	− 2.985
**C-27**	− 38.841	− 3.843	− 41.563	− 4.976
Std 1	− 30.501	− 4.962	− 43.814	− 5.436
Std 2	− 35.263	− 4.223	− 45.831	− 3.451
Std 3	− 31.056	− 5.216	− 31.967	− 5.436
Std 4	− 30.321	− 2.681	− 32.956	− 2.984

*Std 1* cefadroxil,* Std 2* cefotaxime,* Std 3* ciprofloxacin,* Std 4* trimethoprim

**Fig. 4:**
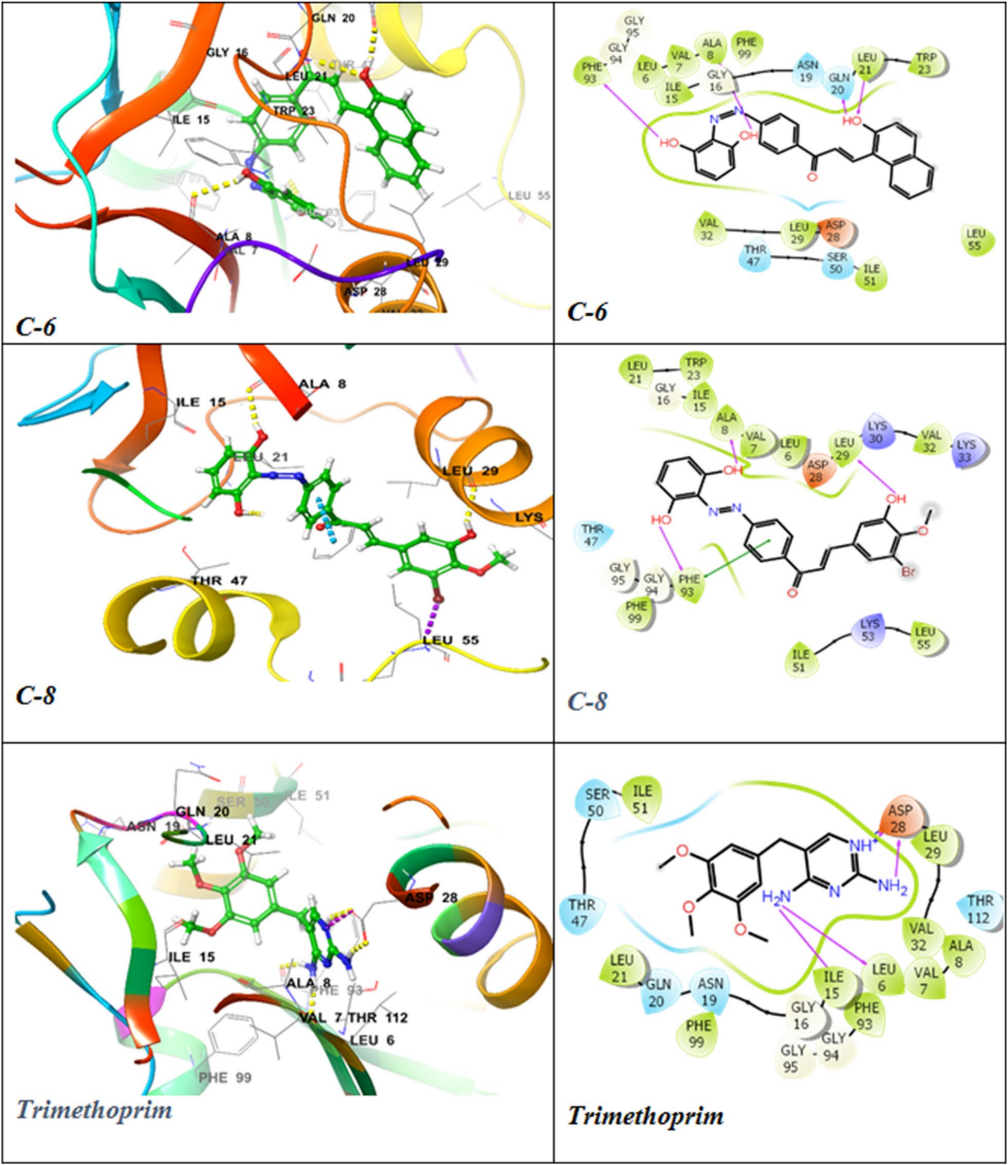
Docked poses and ligand interaction diagram of **C-6**, **C-8** and trimethoprim against *S. aureus* dihydrofolate reductase (PDB ID: 3SRW)

**Fig. 5:**
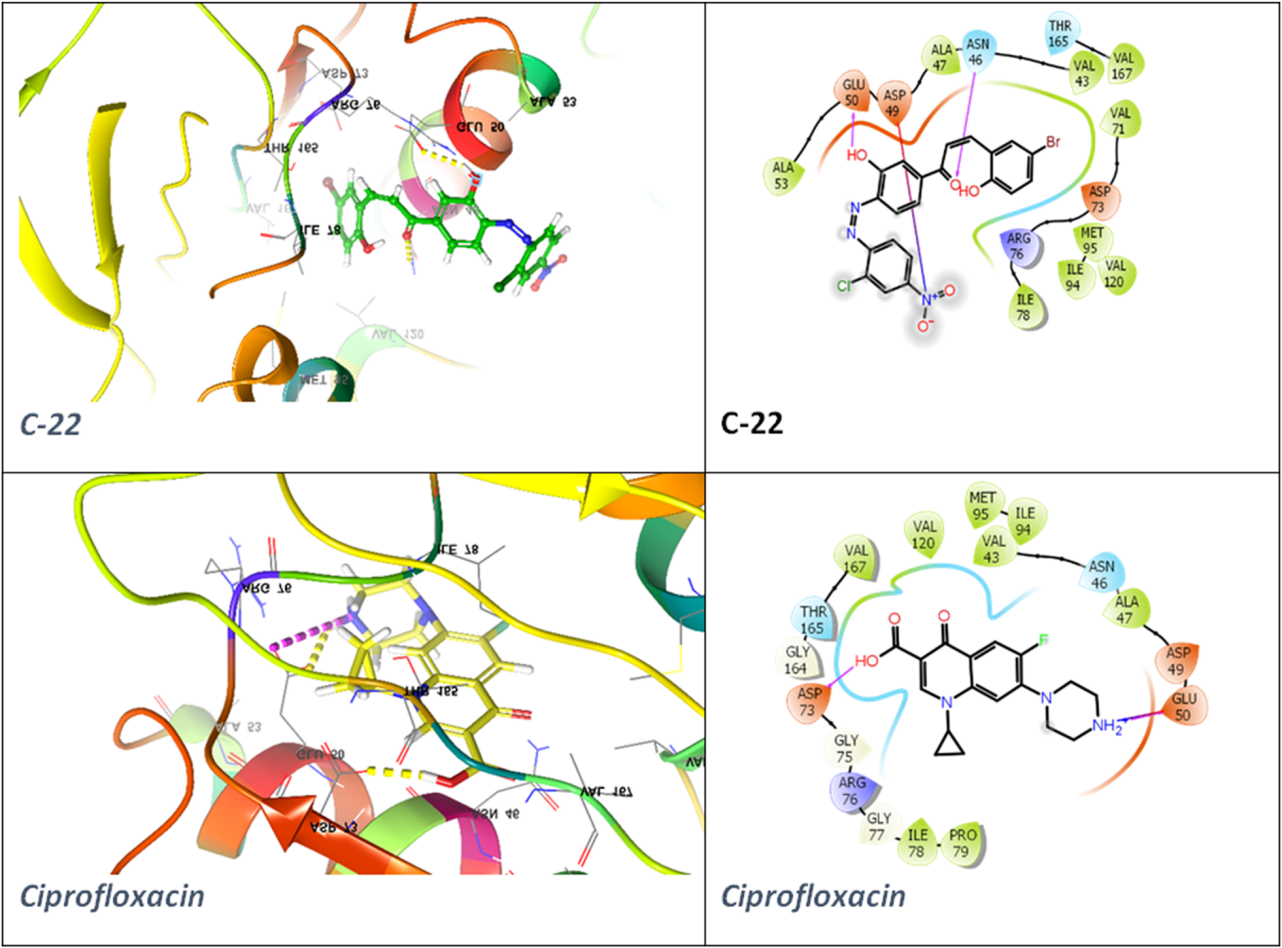
Docked poses and ligand interaction diagram of **C-22** and Ciprofloxacin against DNA gyrase B from *E. coli* (PDB ID: 4ZVI)

**Fig. 6:**
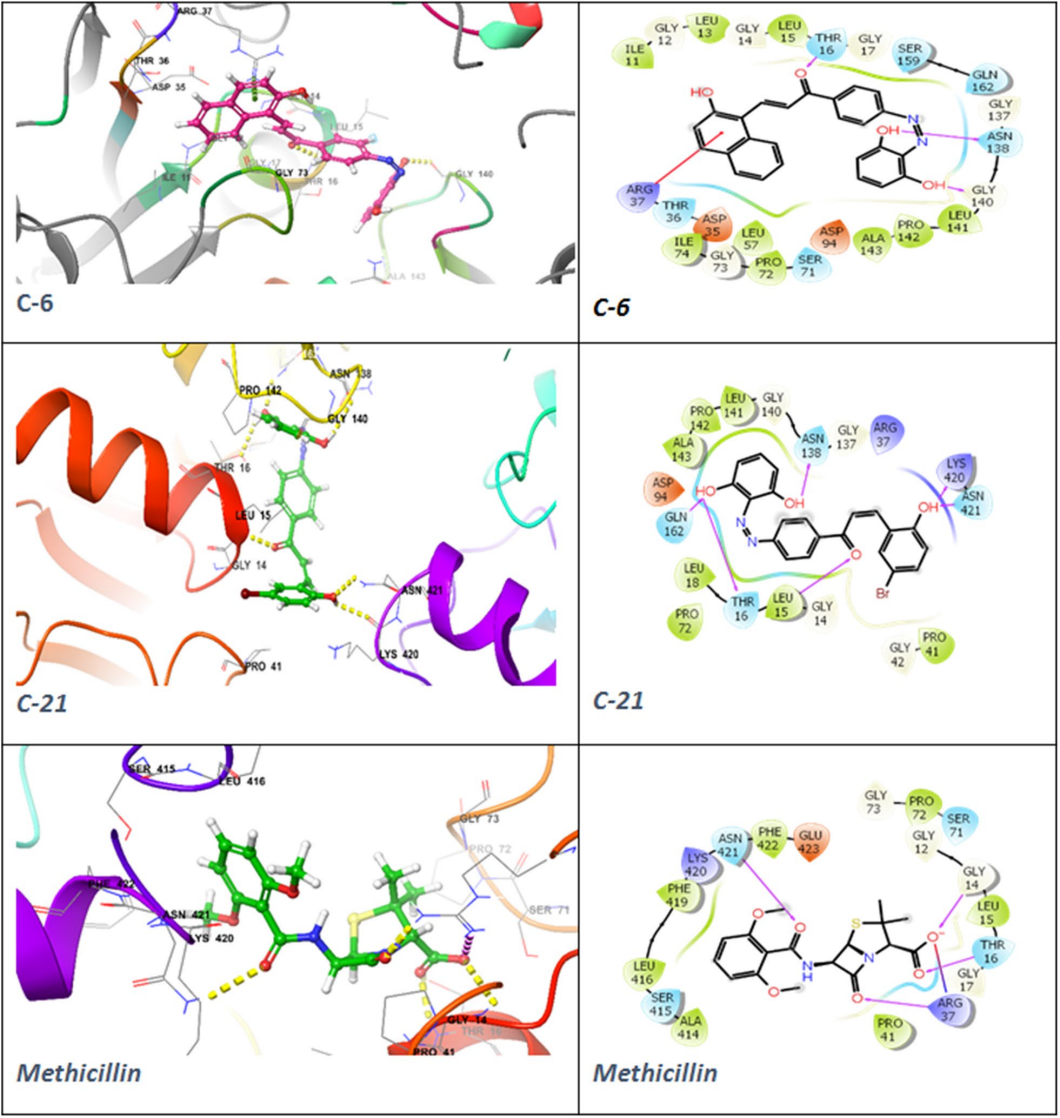
Docked poses and ligand interaction diagram of **C-6**, **C-21** and methicillin against UDP-*N*-acetylmuramoyl-l-alanine:d-glutamate ligase (PDB:1UAG)

The docking poses were visualized for the active derivatives, and it was noticed that the *H*-bonding was the most predominant interactions in the active derivatives. The derivative **C-8** exhibited three hydrogen bonds through –OH groups with the essential residues: Ala8 (2.24 Å), Phe93 (1.65 Å), Leu29 (2.16 Å) of the binding pocket of dihydrofolate reductase (PDB ID: 3SRW). The π–π stacking was also observed for the aromatic ring with the Phe93 (4.33 Å) residue. Similarly, **C-6** derivative exhibited four hydrogen bonds through the –OH groups with the essential residues Leu21 (2.28 Å), Glu20 (1.59 Å), Ala8 (2.29 Å), Phe93 (2.06 Å) while the standard drug trimethoprim exhibited three H-bonds, two donor bonds through amino groups with the Asp28 (2.48 Å) and Ala8 (1.90 Å) residues and one acceptor bond through the pyridyl nitrogen with the Ala8 (2.09 Å) residue of the binding pocket. The ligand interaction diagram and interacting residues of the binding pocket of dihydrofolate reductase with the active derivatives and standard drug trimethoprim had been shown in Fig. 4 and Table 7, respectively. The derivative **C-22** had shown two H-bonds, one through –OH groups with the essential residues Glu50 (2.18 Å) and another H-bond through the > C=O of the enone moiety with the Asn (2.00 Å) of the binding cavity of the DNA gyrase (PDB ID: 4ZVI). The derivative **C-22** also exhibited one salt bridge (3.27 Å) through the NO_2_ group with the Asp49 residue. The standard drug ciprofloxacin displayed two H-bonds one through –OH group with the Asp73 (1.82 Å) and one through the piperazine nitrogen with the Glu50 (2.19 Å) and exhibited one salt bridge (4.48 Å) through the piperazine nitrogen with the Glu50 presented in Fig. 5. The interaction of the active derivatives with the vital residues of the catalytic pocket of the DNA gyrase has been presented in Table 8. UDP-*N*-acetylmuramoyl-l-alanine:d-glutamate ligase (MurD) is one of the crucial enzymes which participates in the peptidoglycan biosynthesis of the cell wall and hence presents a possible target to combat bacterial drug-resistance in search of new antibacterial agents [33]. The **C-21** had shown *H*-bonding interaction with the essential residues Asn421 (2.23 Å), Lys420 (2.38 Å), Thr16 (2.05 Å), Gln162 (1.97 Å), Asn138 (2.16 Å) through OH groups and leu15 (1.86 Å) through C=O group of the binding pocket of the MurD ligase (PDB ID: 1UAG). Similarly, **C-6** had shown H-bonding interaction with the essential residues Gly140 (2.35 Å), Asn138 (1.96 Å) of the binding cavity the through OH groups and Thr16 (2.05 Å), C=O group. The π–π stacking (3.66 Å) was observed for the naphthol ring with the Arg37. The standard dug methicillin demonstrated H-bond through amide linkage with Asn421 (2.60 Å), through lactone ring with the Arg37 (1.97 Å) and the carboxylate group with the Thr16 (1.88 Å) and Gly14 (2.66 Å) and one salt bridge with the carboxylate oxygen with the Arg37 presented in Fig. 6 and Table 9. Hence, the synthesized chalcones have shown their potential as dihydrofolate reductase inhibitors, DNA gyrase inhibitor and the cell wall protein synthesis inhibitors.

**Table 7:** The interaction of highly active compounds with binding pocket of bacterial dihydrofolate reductase (PDB ID 3SRW)

Compound	Interacting residues of the binding pocket
**C-6**	Ala8, Val7, Leu6, Phe93, Phe99, Ile15, Asn19, Gln20, Leu21, Trp23
**C-8**	Lys33, Val32, Lys30, Leu29, Asp28, Phe93, Leu6, Val7, Ala8, Ile15, Trp23, Leu21
**C-24**	Val32, Phe93, Leu29, Asp28, Trp23, Leu21, Gln20, Asn19,Ile15, Thr47,Ser50, Ile51
**C-25**	Leu6, Val7, Ala8, Phe99, Gln96, Phe93, Thr122, Ile15,Asn19, Gln20, Leu21, Trp23
**C-27**	Leu55, Asp28, Leu29, Val32, Leu6, Val7, Ala8, Phe99, Thr122, Gln96, Phe99, Ile15, Asn19, Gln20, Leu21, Trp23
Trimethoprim	Asp28, Leu29, Val32, Ala8, Val7, Phe93, Leu6, Phe99, Ile15, Asn19, Gln20, Leu21

**Table 8:** The interaction of highly active compounds with binding pocket of bacterial DNA gyrase (PDB ID: 4ZVI)

Compound	Interacting residues of the binding pocket
**C-16**	Ala90, Va93, Ile94, Met95, Arg136, Val43, Asn46, Ala47, Asp49, Glu50, Asp73, Arg76, Gly77, Ile78, Pro79
**C-22**	Ala53, Glu50, Asp49, Ala47, Asn46, Val43, Met95, Ile94
Ciprofloxacin	Glu50, Asp49, Ala47, Asn46, Val43, Val167, Thr165, Asp73, Arg76, Ile78, Pro79

**Table 9:** The interaction of highly active compounds with binding pocket of *N*-acetylmuramoyl-l-alanine:D-glutamate ligase (PDB ID:1UAG)

Compound	Interacting residues of the binding pocket
**C-6**	Leu15, Thr16, Asp94, Ala43, Pro142, Leu141, Gly140, Asn138, Gly137, Ser159, Gln162, Arg37, Thr36, Asp35, Ser71, Pro72, Ile74
**C-15**	Ser71, Pro72, Ile74, Arg37, Thr36, Asp35, Ile11, Gly12, Leu15, Thr16, Gly17, Glu423, Phe422, Asn421
**C-21**	Pro41, Gly42, Leu15, Thr16, Leu141, Asn138, Asp94, Gln162, Pro72, Lys420, Asn421
Methicillin	Ala414, Ser415, Leu416, Phe419, Lys420, Asn421, Phe422, Glu423, Pro72, Ser71, Thr16, Leu15, Gly14, Arg37, Pro41

### ADME properties

Most of the newly designed molecules having selectivity and optimized binding affinity for their receptors generally failed during the clinical trials due to deficient pharmacokinetic parameters. Therefore, the screening of promising molecules should not only be restricted to their improved selectivity and increased binding affinity with their targets; but also require significant consideration to the pharmacokinetic parameters such as lipophilicity, blood–brain barrier coefficient, oral bioavailability etc. The QikProp, version 3.5, Schrödinger was used for the prediction of the ADME parameters in comparison to those of 95% known drugs. Total forty-four significant descriptors were predicted for the substituted diazenyl chalcones. The selected descriptors critical for defining the drug-like properties of the synthesized compounds have been reported in Table 10. The Lipinski rule is an important filter which predicts the druggability of the compounds. This rule has been followed by most of the compounds with MW < 500 Da, H-bond acceptor < 10 and donor < 5 and octanol/water partition coefficient (QPlogPo/w) < 5, The synthesized derivatives exhibited oral absorption in the range of 80–100%.

**Table 10:** ADME properties of active diazenyl chalcones by Qikprop module of Schrodinger

Comp	MW	Donor HB	Accept-or HB	QLog Po/w	(QPlogS)	(QPPMDCK)	(QPlogBB)	Rule of five	%Human oral absorption
**C-6**	410.428	3.0	6.250	3.424	− 5.200	48.895	− 2.103	0	84.043
**C-7**	413.259	2.000	5.500	4.115	− 5.812	662.002	− 1.292	0	94.697
**C-8**	469.291	3.000	7.000	3.140	− 5.066	104.407	− 2.035	0	81.688
**C-15**	482.752	1.00	5.750	5.442	− 7.504	1847.029	− 1.013	1	92.715
**C-17**	476.256	1.00	5.750	3.998	− 4.603	613.144	− 1.014	0	93.284
**C-20**	442.257	0.000	4.750	4.610	− 5.615	439.398	− 1.300	0	100
**C-21**	439.264	3.00	6.250	3.109	− 4.857	155.832	− 1.777	0	83.571
**C-22**	502.708	2.00	6.500	3.457	− 5.135	102.524	− 1.935	1	66.001
**C-23**	372.379	2.000	7.500	2.262	− 4.182	27.357	− 2.289	0	73.064
**C-24**	334.331	2.000	6.000	2.527	− 3.692	121.717	− 1.501	0	85.351
**C-25**	425.270	0.000	5.750	0.557	− 3.730	323.257	− 1.148	0	91.261
**C-27**	390.395	2.000	6.000	3.417	− 4.630	114.038	− 1.731	0	90.094
Std 1	363.387	4.250	8.000	− 1.832	− 2.284	1.079	− 2.092	0	13.980
Std 2	455.460	3.250	12.950	0.553	− 4.665	1.772	− 3.696	1	20.880
Std 3	331.346	1.000	6.000	0.280	− 3.792	9.469	− 0.689	0	48.864
Std 4	290.321	4.000	5.250	0.922	− 2.863	131.324	− 1.322	0	76.498

*Std 1* cefadroxil,* Std 2* cefotaxime,* Std 3* ciprofloxacin,* Std 4* trimethoprim,* MW* molecular weight,* Donor HB* hydrogen bond donor,* Acceptor HB* hydrogen bond acceptor,* P*_*O/W*_ partition coefficient in oil and water,* QPlogS* aqueous solubility,* QPPMDCK* apparent MDCK cell permeability,* QPlogBB* brain/blood partition coefficient

## Conclusion

In this investigation, novel diazenyl chalcones (**C18**–**C27**) with various substituted aromatic or heteroaromatic rings were effectively synthesized. These chalcone analogues were evaluated for their in vitro antimicrobial, antioxidant and cytotoxic potential. The results revealed that the chalcone derivative **C-22** was the most active antibacterial agent among all the derivatives by exhibiting very low MIC and MBC. The derivatives with substituted resorcinol ring had shown good antioxidant potential. The derivative **C-25** had shown high selectivity index in comparison to camptothecin against A549 cell line. Further, molecular docking studies predicted that the possible site of interaction of most of the antibacterial derivatives could be the bacterial dihydrofolate reductase while **C-22** can be developed as a novel antimicrobial agent as potent bacterial DNA gyrase inhibitor.

## Experimental

### Materials and methods

The procurement of all the chemicals/reagents required for the study was done from Merck Chemicals (India) and Hi-Media Laboratories (India). The required strains of microorganisms were procured from IMTECH, Chandigarh. The progress of the chemical reaction was monitored using TLC performed with pre-coated plates of silica gel (60 F_254_). The purification of the diazenyl chalcones was done by recrystallization techniques and chromatography by column composed of silica gel of mesh size 100–200, using solvents ethyl acetate and hexane. The infrared spectroscopy (Bruker 12060280, the spectrophotometer) was performed to confirm the presence of functional groups in diazenyl chalcones. The NMR spectroscopy (Bruker Avance II 400 NMR spectrometer), namely ^1^H NMR and ^13^C NMR was carried out to estimate the carbon/proton signals, in DMSO (deuterated) at a frequency of 400 MHz and 100 MHz, respectively. The % composition of hydrogen, carbon, nitrogen, oxygen and sulphur of the synthesized chalcones was estimated on the elemental analyzer (Flash EA1112N series, Thermo Finnigan, Italy). The mass spectroscopy was carried out on the Advion expression CMS, USA mass spectrometer to confirm the molecular mass of the synthesized compounds.

### Synthesis of diazenyl chalcones (**C18**–**C27**)

A solution (10 ml) of substituted anilines (0.01 M) was prepared in 0.2 N HCl. Subsequently, a cold solution of NaNO_2_ (0.01 M) in H_2_O over 10–15 min at 0 °C to form the diazonium salt solution. This solution was stirred at 4–5 °C for half an hour to complete the diazotization and then added dropwise to the ice-cold solution of hydroxy substituted acetophenone derivative in ethanol at 0–5 °C in 30 min. The solution was additionally stirred for 1 h at 4–5 °C. Afterwards, NaOH (10%) solution was added dropwise to neutralize the acid for the precipitation of the azo dyes (**A**–**D**). The azo dye **E** was synthesized in the same manner by diazotization of *p*-aminoacetophenone followed by coupling with resorcinol in an alkaline solution. The resultant precipitates of azo dyes were filtered, air-dried and recrystallized from ethanol. These azo dyes (**A**–**E**) were used for the synthesis of diazenyl chalcones (**C18**–**C27**) by reaction with various mono or di-substituted aromatic/heteroaromatic aldehydes in the presence of the catalytic amount of alkali (Scheme 1). In a 250 ml conical flask, 0.001 M of aldehyde and 0.001 M of azo dye were dissolved in the presence of ethanol and stirred vigorously on a magnetic stirrer. After 30 min, 2 ml of 10% alcoholic KOH was added drop-wise to the reaction mixture with rapid stirring keeping the reaction temperature maintained at 25–30 °C with continuous stirring for a period of 18–24 h. The progress of the reaction was monitored by TLC. Afterwards, the contents of the flask were transferred into the ice-chilled water and neutralized by 0.1–0.2 N HCl, resulting in the precipitation of diazenyl chalcone derivatives. The precipitates obtained were air dried after filtration, and subject to purification by recrystallization from ethanol and by column chromatography (solvent system of 5–25% ethyl acetate and hexane) [27].

#### Analytical data

##### 1-(3-((2,3-Dichlorophenyl)diazenyl)-4-hydroxyphenyl)ethanone (**A**)

M.F: C_14_H_10_Cl_2_N_2_O_2_. Mol. Wt: 309.15 g; Orange, Yield = 77%; R_f_ = 0.54 (pet ether/ethyl acetate 5:2); mp: 140–142 °C; IR (KBr, cm^−1^) ν_max_: 3413.85, 3079.52, 3008.26, 2924.69, 2854.77, 1678.55, 1598.85, 1496.35, 1452.21, 1419.45, 1360.09, 1269.13, 1240.13, 958.76, 812.42, 702.96, 572.13.

##### 1-(3-((2,3-Dichlorophenyl)diazenyl)-2-hydroxyphenyl)ethanone **(B)**

M.F: C_14_H_10_Cl_2_N_2_O_2_. Mol. Wt: 309.15 g, Orange color; Yield = 86%; R_f_ = 0.52 (pet ether/ethyl acetate 5:2); mp: 135–138 °C; IR (KBr, cm^−1^) ν_max_: 3418.19, 3065.43, 2973.13, 1649.37, 1570.29, 1484.30, 1416.72, 1368.89, 1322.61, 1292.04, 1256.54, 1202.40, 1168.00, 1117.88, 906.49, 821.90, 788.95, 637.17.

##### 1-(3-((2,5-Dichlorophenyl)diazenyl)-2-hydroxyphenyl)ethanone (**C**)

M.F: C_14_H_10_Cl_2_N_2_O_2_. Mol. Wt: 309.15 g; Brownish red; Yield = 81%; R_f_ = 0.55 (pet ether/ethyl acetate 5:2); mp: 95–98 °C; IR (KBr, cm^−1^) ν_max_: 3420.06, 1638.80, 1483.33, 1371.86, 1292.82, 1217.75, 1085.78, 1055.21, 956.40, 897.51, 810.63, 640.41, 527.08, 456.00.

##### 1-(4-((2-Chloro-4-nitrophenyl)diazenyl)-3-hydroxyphenyl)ethanone (**D**)

M.F: C_14_H_10_ClN_3_O_4_. Mol. Wt: 319.7 g, Red; Yield = 81%; R_f_ = 0.49 (pet ether/ethyl acetate 3:2); mp: 160–163 °C; IR (KBr, cm^−1^) ν_max_: 3353.91, 3101.43, 2926.98, 2460.67, 1652.56, 1586.85, 1522.93, 1460.79, 1414.00, 1345.93, 1230.84, 1177.06, 1119.72, 1047.34, 889.75, 883.20, 839.50, 801.66, 742.88, 699.41.

##### 1-(4-((2,6-Dihydroxyphenyl)diazenyl)phenyl)ethanone (**E**)

M.F: C_14_H_12_N_2_O_3_. Mol. Wt: 256 g; Orange; Yield = 73%; R_f_ = 0.59 (pet ether/ethyl acetate 2:1); mp: 240–242 °C; IR (KBr, cm^−1^) ν_max_: 3248.43, 2821.91, 1657.33, 1595.72, 1483.05, 1418.91, 1353.65, 1250.88, 1199.15, 1177.60, 1033.22, 963.36, 838.60, 742.89, 652.38.

##### (2*E*)-3-(2,4-Dichlorophenyl)-1-(3-((2,3-dichlorophenyl)diazenyl)-4-hydroxyphenyl)prop-2-en-1-one (**C-18**)

Brown, Yield: 63%, R_f_: 0.49 (ethylacetate: hexane; 2:8); mp: 110–112 °C, FTIR (KBr, cm^−1^) ν_max_: 3417.79, 3077.91, 3009.11, 1682.10, 1598.86, 1562. 54, 1497.93, 1412.43, 1412. 08, 1360. 89, 1277.17, 1270.28, 1240.12, 1134.80, 1101.61, 1070.37, 1049.40, 986.10, 740.02, 678.03, 575.02; ^1^H NMR (400 MHz, DMSO-d_6_) δ: 11.88 (s, 1H), 8.33 (d, *J *= 2.0 Hz, 1H), 8.29 (d, *J *= 2.0 Hz, 1H), 8.1 (s, 1H), 8.08 (dd, *J*_*1*_= 8.8 Hz, *J*_*2*_= 2.0 Hz, 1H), 7.84–7.89 (m, 3H), 7.55 (t, *J *= 8.0 Hz, 1H), 7.68 (d, *J *= 8.4 Hz, 1H), 7.47 (dd, *J*_*1*_= 8.4 Hz, *J*_*2*_= 2.0 Hz, 1H), 7.21 (d, *J *= 8.4 Hz, 1H); ^13^C NMR (100 MHz, DMSO-d_6_) δ: 196.50, 159.56, 149.40, 138.72, 134.72, 133.45, 133.20, 132.23, 129.56, 129.19, 127.98, 123.06, 119.14, 117.26; APCI-MS m/z found for C_21_H_12_Cl_4_N_2_O_2_: 466 (M^+^); Anal. calcd for C_21_H_12_Cl_4_N_2_O_2_: C 54.11, H 2.59, Cl 30.42, N 6.01, O 6.86 found: C 54.13, H 2.62, N 6.03, O 6.89.

##### (2*Z*)-3-(2,4-Dichlorophenyl)-1-(3-((2,3-dichlorophenyl)diazenyl)-2-hydroxyphenyl)prop-2-en-1-one (**C-19**)

Brown, Yield: 69%, R_f_: 0.32 (ethylacetate: hexane; 3:7), mp: 112–114 °C, (KBr, cm^−1^) ν_max_: 3447.43, 1639.24, 1584.20, 1480.35, 1414.57, 1370.31, 1268.20, 1193.72, 1160.09, 1112.06, 1073.33, 1049.39, 817.67, 788.45, 743.08, 531.06; ^1^H NMR (400 MHz, DMSO-d_6_) δ: 12.19 (s, 1H), 8.44 (d, *J *= 2.0 Hz, 1H), 8.08 (dd, *J*_*1*_= 8.8 Hz, *J*_*2*_= 2.0 Hz, 1H), 7.81 (d, *J *= 8.0 Hz, 1H), 7.69 (d, *J *= 8.4 Hz, 1H), 7.46–7.62 (m, 4H), 7.21 (d, *J *= 8.8 Hz, 1H), 5.76 (s, 1H), 5.50–5.52 (m, 1H); ^13^C NMR (400 MHz, DMSO-d_6_) δ: 192.8, 157.0, 154.3, 145.1, 136.0, 132.4, 131.3, 130.3, 130.2, 130.1, 128.9, 128.5, 126.8, 125.6, 125.2, 122.9, 122.5, 122.1, 121.3, 116.9; APCI-MS m/z found for C_21_H_12_Cl_4_N_2_O_2_: 466 (M^+^); Anal. calcd for C_21_H_12_Cl_4_N_2_O_2_: C 54.11, H 2.59, Cl 30.42, N 6.01, O 6.86 found: C 54.10, H 2.61, N 6.03, O 6.88.

##### (2*Z*)-1-(3-((2,3-Dichlorophenyl)diazenyl)-2-hydroxyphenyl)-3-(2-nitrophenyl)prop-2-en-1-one (**C-20**)

Brown, Yield: 61%, R_f_: 0.31 (ethylacetate: hexane; 3:7); mp: 105–107 °C; FTIR (KBr, cm^−1^) ν_max_: 3571.48, 3419.29, 3075.18, 1640.43, 1578.61, 1525.31, 1482.79, 1415.73, 1358.51, 1275.44, 1192.07, 1163.17, 1053.73, 1024.77, 786.94, 745.58; ^1^H NMR (400 MHz, DMSO-d_6_) δ: 12.36 (s, 1H), 8.67 (d, *J *= 2.4 Hz, 1H), 8.44–8.46 (m, 1H), 8.06–8.08 (m, 1H), 7.91–7.99 (m, 2H), 7.81 (dd, *J*_*1*_= 7.6 Hz, *J*_*2*_= 1.6 Hz, 1H), 7.49–7.60 (m, 3H), 7.18–7.21 (m, 2H); ^13^C NMR (400 MHz, DMSO-d_6_) δ: 203.43, 164.13, 150.06, 145.13, 140.42, 133.93, 133.43, 132.55, 131.93, 130.02, 129.14, 128.87, 128.09, 124.36, 122.14, 119.58, 116.77; APCI-MS m/z found for C_21_H_13_Cl_2_N_3_O_4_: 442 (M^+^); Anal. calcd for C_21_H_13_Cl_2_N_3_O_4_: C 57.03, H 2.96, Cl 16.03, N 9.50, O 14.47 found: C 57.04, H 3.00, N 9.52, O 14.48.

##### (2*Z*)-3-(5-Bromo-2-hydroxyphenyl)-1-(4-((2,6-dihydroxyphenyl)diazenyl)phenyl)prop-2-en-1-one **(C-21)**

Orange, Yield: 69%, R_f_: 0.54 (ethylacetate: hexane; 7:2); mp: 155–157 °C; (KBr, cm^−1^) ν_max_: 3383.58, 2304.81, 1949.24, 1628.99, 1593.80, 1526.40, 1496.43, 1415.68, 1355.42, 1315.57, 1222.61, 1154.77, 1117.86, 1066.46, 1032.14, 1007.24, 835.51, 754.90, 681.00; ^1^H NMR (400 MHz, DMSO-d_6_) δ: 12.36 (s, 1H), 10.76 (s, 1H), 9.47 (s, 1H), 8.25 (d, *J *= 8.4 Hz, 1H), 8.10 (d, *J *= 8.8 Hz, 2H), 7.91 (d, *J *= 8.4 Hz, 1H), 7.79 (d, *J *= 7.6 Hz, 1H), 7.41–7.73 (m, 5H), 6.51–6.55 (m, 1H), 6.37–6.39 (m, 1H); ^13^C NMR (100 MHz, DMSO-d_6_) δ: 197.74, 164.61, 158.18, 153.80, 139.37, 137.51, 133.45, 133.33, 132.43, 130.87, 129.84, 127.84, 122.31, 119.28, 118.36, 110.22, 103.52, 67.66; APCI-MS m/z found for C_21_H_15_BrN_2_O_4_: 439 (M^+^); Anal. calcd for C_21_H_15_BrN_2_O_4_: C 57.42, H 3.44, Br 18.19, N 6.38, O 14.57 found: C 57.46, H 3.46, N 6.40, O 14.59.

##### (2Z)-3-(5-Bromo-2-hydroxyphenyl)-1-(4-((2-chloro-4-nitrophenyl)diazenyl)-3-hydroxyphenyl) prop-2-en-1-one **(C-22)**

Brown, Yield: 72%, R_f_: 0.45 (ethylacetate: hexane; 3:7) mp: 160–162 °C; FTIR (KBr, cm^−1^) ν_max_: 3369.21, 3098.89, 2925.24, 2856.93, 1659.29, 1584.80, 1521.69, 1459.82, 1407.56, 1345.57, 1229.66, 1174.28, 1117.33, 1046.62, 989.17, 886.74, 839.95, 742.62, 700.72, 632.03; ^1^H NMR (400 MHz, DMSO-d_6_) δ: 11.06 (s, 1H), 10.20 (s,1H), 8.43 (d, *J *= 2.0 Hz, 1H), 8.28 (dd, *J*_*1*_= 9.2 Hz, *J*_*2*_= 2.4 Hz), 7.83 (d, *J *= 8.8 Hz, 1H), 7.7–7.73 (m, 3H), 7.64 (dd, *J*_*1*_= 8.8 Hz, *J*_*2*_= 2.4 Hz), 7.07 (dd, *J*_*1*_= 8.8 Hz, *J*_*2*_= 2.4 Hz), 6.95–6.98 (m, 2H); ^13^C NMR (400 MHz, DMSO-d_6_): 201.76, 189.58, 162.57, 159.82, 151.37, 148.17, 142.41, 142.20, 138.43, 133.52, 130.38, 125.91, 123.96, 123.58, 122.26, 119.85, 118.74, 118.10, 113.94, 110.67; APCI-MS m/z found for C_21_H_13_BrClN_3_O_5_: 502.70 (M^+^); Anal. calcd for C_21_H_13_BrClN_3_O_5_: C 50.17, H 2.16, Br 15.89, Cl 7.05, N 8.36, O 15.91 found: C 50.20, H 2.19, N 8.38, O 15.95.

##### 4-((1Z)-3-(4-((2,6-Dihydroxyphenyl)diazenyl)phenyl)-3-oxoprop-1-en-1-yl)benzaldehyde**: (C-23)**

Dark red, Yield: 69%, R_f_: 0.22 (ethylacetate: hexane; 7:3); mp: 182–184 °C; FTIR (KBr, cm^−1^) ν_max_: 3061.53, 2916.32, 1678.00, 1600.42, 1496.68, 1450.84, 1358.79, 1260.68, 1137.13, 1015.34, 958.09, 833.35, 717.76, 596.86, 502.44; ^1^H NMR (400 MHz, DMSO-d_6_): 13.93 (s, 1H), 11.01 (s, 1H), 9.94 (s, 1H), 8.04–8.10 (m, 4H), 7.75–7.95 (m, 4H), 7.60–7.67 (m, 2H), 7.32 (d, *J *= 8.0 Hz, 1H), 6.60–6.63 (m, 1H), 6.50 (s, 1H); ^13^C NMR (400 MHz, DMSO-d_6_) δ: 197.05, 192.56, 159.94, 151.48, 139.60, 136.31, 133.62, 132.65, 130.40, 129.97, 129.64, 129.27, 128.28, 121.29, 120.66, 115.22; APCI-MS m/z found for C_22_H_16_N_2_O_4_: 372 (M^+^); Anal. calcd for C_22_H_16_N_2_O_4_: C70.96, H 4.33, N 7.52, O 17.19 found: C 70.96, H 4.30, N 7.49, O 17.17.

##### (2*Z*)-1-(4-((2,6-Dihydroxyphenyl)diazenyl)phenyl)-3-(furan-2-yl)prop-2-en-1-one (**C-24**)

Dark red, Yield: 66%, R_f_: 0.22 (ethylacetate: hexane; 5:5); mp: 315–317 °C; FTIR (KBr, cm^−1^) ν_max_:3434.48, 1673.36, 1596.74, 1497.34, 1451.52, 1261.80, 1136.30, 1011.52, 958.72, 832.20, 718.28, 593.81; ^1^H NMR (400 MHz, DMSO-d_6_) δ: 13.95 (s, 1H), 10.89 (s, 1H), 8.06 (d, *J *= 7.6 Hz, 2H), 7.91 (d, *J *= 8.4 Hz, 2H), 7.59 (d, *J *= 8.4 Hz, 2H), 7.50 (s, 1H), 6.6 (d, *J *= 8.8 Hz, 2H), 6.30–6.32 (m, 2H), 5.81 (d, *J *= 2.0 Hz, 1H); ^13^C NMR (400 MHz, DMSO-d_6_) δ: 197.07, 159.90, 154.60, 151.34, 136.32, 132.61, 130.13, 129.66, 120.92, 120.64, 114.32, 110.38, 105.35; APCI-MS m/z found for C_19_H_14_N_2_O_4_: 334.33 (M^+^); Anal. calcd for C_19_H_14_N_2_O_4_: C 68.26, H 4.22, N 8.38, O 19.14 found: C 68.24, H 4.19, N 8.36, O 19.16.

##### 4-((1*Z*)-3-(3-((2,3-Dichlorophenyl)diazenyl)-2-hydroxyphenyl)-3-oxoprop-1-en-1-yl)benzaldehyde (**C-25**)

Dark red, Yield: 75%, R_f_: 0.52 (ethylacetate: hexane; 5:5); mp: 96–98 °C; (KBr, cm^−1^) ν_max_: 3456.51, 1685.36, 1644.48, 1606.76, 1538, 1460.04, 1351.19, 1280.89, 1164.34, 1042.12, 960.34, 857.34, 789.45, 651.23; ^1^H NMR (400 MHz, DMSO-d_6_) δ: 12.33 (s, 1H), 9.99 (s, 1H), 8.42 (d, *J *= 2.4 Hz, 1H), 8.06 (dd, *J*_*1*_= 8.8 Hz, *J*_*2*_= 2.4 Hz, 1H), 7.89–7.93 (m, 2H), 7.79–7.82 (m, 2H), 7.61–7.68 (m, 2H), 7.49–7.57 (s, 1H), 7.20 (d, *J *= 9.2 Hz, 1H), 5.67–5.72 (m, 1H), 5.27–5.30 (m, 1H); ^13^C NMR (400 MHz, DMSO-d_6_) δ: 192.77, 183.21, 152.11, 149.59, 144.73, 135.17, 132.91, 132.03, 131.41, 129.50, 128.65, 127.91, 126.56, 122.76, 119.04, 116.31; APCI-MS m/z found for C_22_H_14_Cl_2_N_2_O_3_: 425 (M^+^); Anal. calcd for C_22_H_14_Cl_2_N_2_O_3_: C 62.13, H 3.32, Cl 16.67, N 6.59, O 11.29 found: C 62.15, H 3.36, N 6.61, O 11.32.

##### (2Z)-3-(3-Bromophenyl)-1-(3-((2,5-dichlorophenyl)diazenyl)-2-hydroxyphenyl)prop-2-en-1-one (**C-26**)

Brown, Yield: 67%, R_f_: 0.45 (hexane:ethyacetate:6:4); mp: 140–142 °C; FTIR (KBr, cm^−1^) ν_max_: 3310.82, 3104.32, 3012.83, 2974.14, 1677.80, 1592.96, 1503.49, 1461.04, 1427.59, 1353.94, 1295.26, 1165.18, 1042.31, 971.21, 857.20, 825.15, 788.77, 726.06, 677.18; ^1^H NMR (400 MHz, DMSO-d_6_) δ: 12.31 (s, 1H), 8.50 (d, *J *= 2.8 Hz, 1H), 8.07 (dd, *J *= 8.8 Hz, J2 = 2.4 Hz, 1H), 7.81 (d, *J *= 8.8 Hz, 1H), 7.71 (d, *J *= 8.8 Hz, 1H), 7.61–7.64 (m, 2H), 7.29–7.46 (m, 2H), 7.20 (d, *J *= 8.8 Hz, 1H), 5.63 (d, *J *= 4 Hz, 1H), 5.19 (d, *J *= 4 Hz, 1H); ^13^C NMR (400 MHz, DMSO-d_6_) δ: 202.64, 163.81, 148.86, 148.50, 145.13, 133.26, 132.74, 132.66, 131.99, 130.83, 130.30, 129.89, 129.17, 128.08, 125.48, 122.98, 122.04, 119.49, 117.86, 68.97; APCI-MS m/z found for C_21_H_13_BrCl_2_N_2_O_2_: 476 (M^+^); Anal. calcd for C_21_H_13_BrCl_2_N_2_O_2_: C 52.97, H 2.75, Br 16.78, Cl 14.89, N 5.88, O 6.72, found: C 52.94, H 2.78, N 5.89, O 6.75.

##### (2Z)-1-(4-((2,6-Dihydroxyphenyl)diazenyl)phenyl)-3-(2-methoxyphenyl)prop-2-en-1-one (**C-27**)

Red, Yield: 63%, R_f_: 0.37 (hexane: ethylacetate: ethanol; 3:5:2) mp: 320–322 °C; FTIR (KBr, cm^−1^) ν_max_: 3421.26, 3064.94, 2921.26, 1679.07, 1598.74, 1496.44, 1453.23, 1359.03, 1264.76, 1137.13, 1016.93, 958.50, 718.16, 665.27, 590.08; ^1^H NMR (400 MHz, DMSO-d_6_) δ:14.19 (s, 1H), 12.2 (s, 1H), 8.04 (d, *J *= 8.8 Hz, 2H), 7.78 (d, *J *= 8.4 Hz, 2H), 7.34 (d, *J *= 8.4 Hz, 1H), 7.06–7.11 (m, 1H), 6.75–6.84 (m, 2H), 6.56 (s, 1H), 6.37 (d, *J *= 7.6 Hz, 1H), 3.54 (s, 3H); ^13^C NMR (400 MHz, DMSO-d_6_) δ: 189.7, 159.2, 158.5, 153.6, 141.0, 140.1, 133.7, 130.2, 123.5, 121.3, 125.7, 135.2, 128.9, 120.9, 114.2, 56.2; APCI-MS m/z found for C_22_H_18_N_2_O_4_: 374 (M^+^), Anal. calcd for C_22_H_18_N_2_O_4_: C 70.58, H 4.85, N 4.48, O 17.09 found: C 70.55, H 4.88, N 4.50, O 17.11.

### Biological assays

#### Microorganisms

The microbial strains used in the study were Gram-positive bacteria: *Staphylococcus aureus* MTCC 2901; *Bacillus subtilis* MTCC 2063, *Bacillus cereus* MTCC 1305, Gram-negative bacterial: *Escherichia coli* MTCC 1652, *Salmonella typhi* MTCC 3216, and fungal strains: *Aspergillus fumigatus* MTCC 2584 and *Candida albicans* MTCC 227.

#### Determination of MIC and MBC/MFC values

The synthesized diazenyl chalcones (**C18** to **C27**) and controls (dyes A and E, and chalcone) were assessed for their antimicrobial activity by estimation of MIC and MBC/MFC values [34, 35]. The cefotaxime, cefadroxil, SXT, CIP and fluconazole were used as reference drugs. The stock solutions of the test compounds and standard drugs having a concentration of 1000 µg ml^−1^ were prepared by dissolving the used compounds in DMSO. The nutrient broth/Sabouraud dextrose broth was used for the serial dilution of the test compounds and referenced drugs for bacterial and fungal cultures, respectively. The various concentrations prepared were; 500, 250, 125, 62.5, 31.25, 15.62, 7.81, 3.90, 1.95 μg ml^−1^ under sterile conditions. The size of the final inoculum in each test tube was taken as 5 × 10^5^ CFU ml^−1^. The incubation of the test and standard compounds of varying concentrations was done for 24 h at 37 ± 2 °C (bacterial culture) and for 48 h at 37 ± 2 °C (*C. albicans*) and for 7 d at 25 ± 2 °C (*A. fumigatus*). The diazenyl chalcones (**C18**–**C27**), were further evaluated for their bactericidal/fungicidal activity by estimation of MBC/MFC values. 100 µl sample was transferred from the test tube exhibiting absence of growth in MIC experiment, aseptically to the sterilised petri plates followed by the addition of 15–20 ml of nutrient media. For proper mixing of culture media, the plates were shaken gently followed by incubation at the predefined temperature and time as stated earlier, followed by the visual inspection of the plates for signs of microbial growth. The MBC/MFC values represent the minimum concentration of test compounds in petri plate, which showed no visual sign of growth after incubation.

#### Antioxidant evaluation

The derivatives (**C18**–**C27**) were assessed for their antioxidant potential in the presence of the stable free radical DPPH using ascorbic acid as the standard compound [36, 37]. The assay was performed in 96 well plates using ELISA reader with samples aliquots at a series of concentrations of 1.56, 3.12, 6.25, 12.5, 25, 50 and 100 µg ml^−1^ in methanol in different wells. The total volume of 200 µl was used in each well for each concentration of the test and standard drug containing 0.004 µg DPPH. The assay was performed in triplicate at all concentrations of each sample and standard compound. DPPH solutions in methanol at the same concentration without test drugs were used as the control. Each concentration of the sample and standard has a different blank. To minimize evaporation, the plate was covered with the lid and wrapped in aluminium foil and kept in the dark place in order to protect the DPPH radical from degradation by light and left for 30 min. After 30 min of incubation, the plate was kept in ELISA reader to measure the absorbance values at 517 nm. The experiment was performed in triplicate. The antioxidant activity of each sample was then calculated as per cent inhibition of DPPH according to the following equation:

$$\% \;{\text{decolouration}}\;{\text{of}}\;{\text{DPPH}} = \left( {{\text{Control}}_{\text{Abs}} - {\text{Sample}}_{\text{Abs}} } \right) \times 100/{\text{Control}}_{\text{Abs}}$$

#### Cytotoxicity evaluation

The cell lines employed in the investigation were purchased from the National Centre for Cell Sciences (NCCS), Pune, India. The cell lines were cultured in DMEM fortified with 10% FBS, l-glutamine, sodium bicarbonate, and solution of antibiotics (streptomycin 100 μg ml^−1^ + penicillin-100 U ml^−1^). The culture of cell lines was stored under 5% CO_2_ incubation at 37 °C.

#### Cell proliferation study by MTT assay

The compounds showing maximum activity (antimicrobial and antioxidant potential) were screened for cytotoxicity against A549 and HEK cell lines using MTT assay [38, 39]. Initially, 96-well microculture plate was seeded with 1 × 10^4^ cells in 100 µl/well DMEM/MEM, supplemented with 10% FBS followed by incubation at a temperature of 37 °C for a period of 24 h under 5% CO_2_ atmosphere. After that, the test compounds and camptothecin were added to the cells to achieve a concentration of 10, 25, 50 and 100 µg. MTT reagent (10 µl) of concentration of 5 mg ml^−1^ added to all the wells after the duration of 48 h. The plate was incubated further for 4 h followed by careful removal of supernatant from each well. To dissolve the formazan crystals, 100 µl of DMSO was added, and the ELISA reader was used to check the absorbance at 570 nm wavelength [40]. In order to calculate the IC_50_ concentration, the following equation, i.e. A = Bx + C, was used. In this case, A = 50, while the values B and C values were obtained from the survival curve plot. This study was carried out in duplicate.

#### Molecular docking

The active antimicrobial compounds found from the previous series synthesized by Kaur and Narasimhan (**C-6**, **C-7**, **C-8**, **C-15**, **C-16**, **C-17**) [27] and this series (**C-20**, **C-21**, **C-22**, **C-23**, **C-24**, **C-25** and **C-27**) were docked for the various potential bacterial targets such as dihydrofolate reductase, dihydropteroate synthase, cell wall synthesis proteins, DNA gyrase and topoisomerase using Schrodinger Glide software [41]. The 3-dimensional crystal structures of all the proteins such as *S. aureus* dihydrofolate reductase (PDB ID: 3SRW, resolution 1.7 Å), *E. coli* gyrase B (PDB ID: 4ZVI, resolution 2.2 Å), *E. coli* UDP-*N*-acetylmuramoyl-l-alanine:d-glutamate ligase (PDB ID: 1UAG, resolution 1.95 Å), *E. coli* topoisomerase IV (PDB ID: 3FV5, resolution 1.8 Å), and dihydropteroate synthase from *Streptococcus pneumonia (*(PDB ID: 3FV5, resolution 2.4 Å) were used for the molecular modelling and were accessed from the website of Protein Data Bank (http://www.rcsb.org/pdb/home/home) [42]. The derivatives under investigation were studied for the mode of theoretical binding to comprehend the possible intermolecular interactions between the ligand and the receptor. The required protein structures were prepared (preprocessed, optimized and minimized) by the Protein Preparation Wizard available in the Schrodinger software graphical user interface Maestro *v11.5*. Crystallographic H_2_O molecules with one or two H-bonds were omitted. To set the pH of the protein to a value of 7.0, hydrogen atoms were added to the structure. To attain the RMSD cut off 0.30 Å, the restrained minimization of the heavy atoms was performed. The ligands (data set) were prepared using the LigPrep module of Schrodinger *v11.5.* An active site encompassing a radius of 20 Å was defined around the ligand in the crystal structure of the proteins. Also, a grid box was created around the centroid of the defined active site. All the ligands with their low-energy conformations were docked into different proteins in their respective catalytic pockets using extra precision mode (Glide, Schrödinger 2018-1) in the absence of constraints. The structures showing the best results were selected based on docking scores and binding energies [43, 44].

#### ADME prediction

The most prevalent reason for the failure of drugs in clinical trial phase is the lack of knowledge regarding its ADME parameters. Therefore, for any new drug development, the prediction of ADME parameters is the crucial step. The ADME parameters like oral bioavailability, partition coefficient, blood–brain barrier coefficient etc. of the synthesized compounds were determined using QikProp module of the Schrodinger *v11.5*. The ligands were prepared in the LigPrep module in the Maestro format (*.maez*) for the investigation of ADME parameters. Then Ligprep file for the selected ligands was browsed into the Qikpro dialogue box and press the run command to obtain the ADME parameters. The selected parameters were noted from the project table entry file [45].

## Additional file

**Additional file 1.** ^1^H and ^13^C NMR data of most active compounds has been provided.

## Data Availability

Provided as additional file.

## Abbreviations

DMEM: Dulbecco’s Modified Eagle Medium
RMSD: root mean square distance
MEM: Minimum Essential Medium
FBS: fetal bovine serum
MTT: 3-(4,5-dimethylthiazol-2-yl)-2,5-diphenyltetrazolium bromide
MIC: minimum inhibitory concentration
MBC: minimum bactericidal concentration
MFC: minimum fungicidal concentration
ADME: absorption, distribution, metabolism, excretion
ELISA: enzyme-linked immunosorbent assay
HEK-293: human embryonic kidney cells
DPPH: 2,2-diphenyl-1-picrylhydrazyl
SXT: sulfamethoxazole/trimethoprim
CIP: ciprofloxacin
IMTECH: Institute of Microbial Technology and Genebank, Chandigarh
