# Supplementary material for: Antimicrobial, antioxidant and cytotoxic evaluation of diazenyl chalcones along with insights to mechanism of interaction by molecular docking studies
Source: BMC Chem. 2019 Jul 9;13(1):87. doi: 10.1186/s13065-019-0596-5 (PMC6661766; doi:10.1186/s13065-019-0596-5)

**Antimicrobial, antioxidant and cytotoxic evaluation of diazenyl chalcones alongwith insights to mechanism of interaction by molecular docking studies**

Harmeet Kaur^1^, Jasbir Singh^2^, Balasubramanian Narasimhan^1^*

The ^1^H and ^13^C data of the synthesized compounds have been provided.


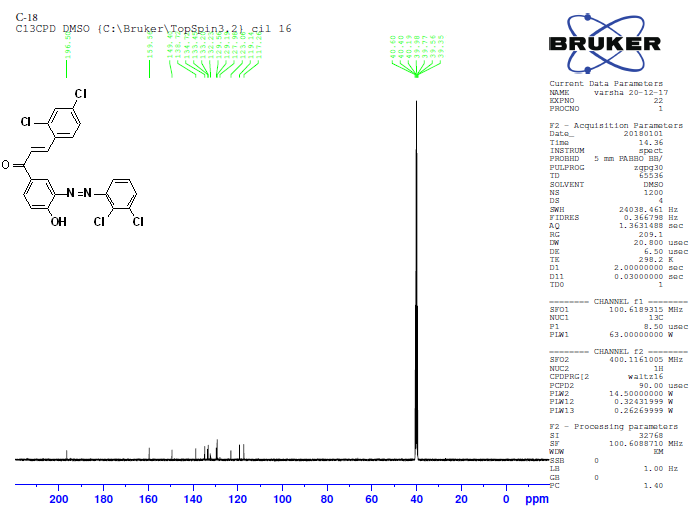


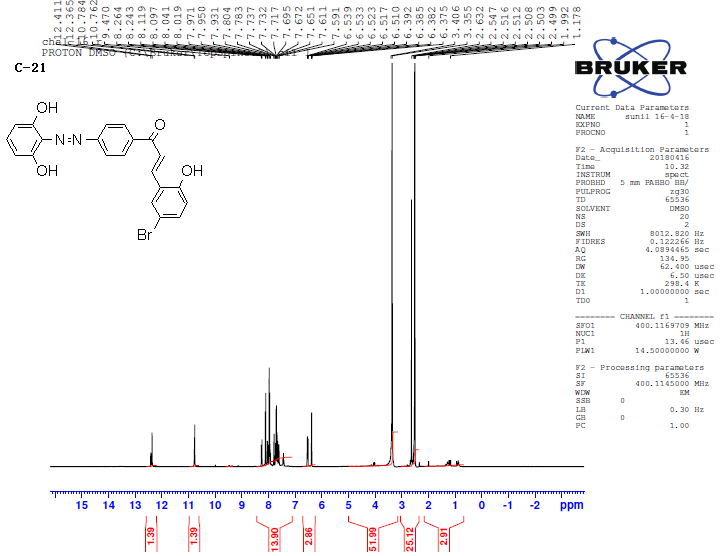

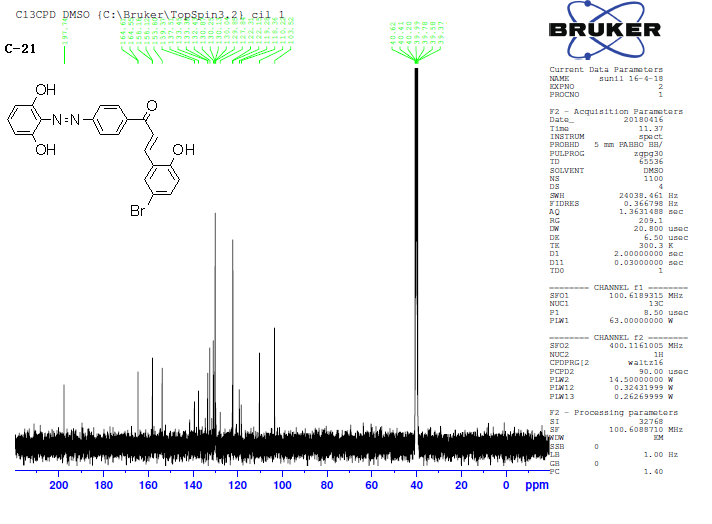

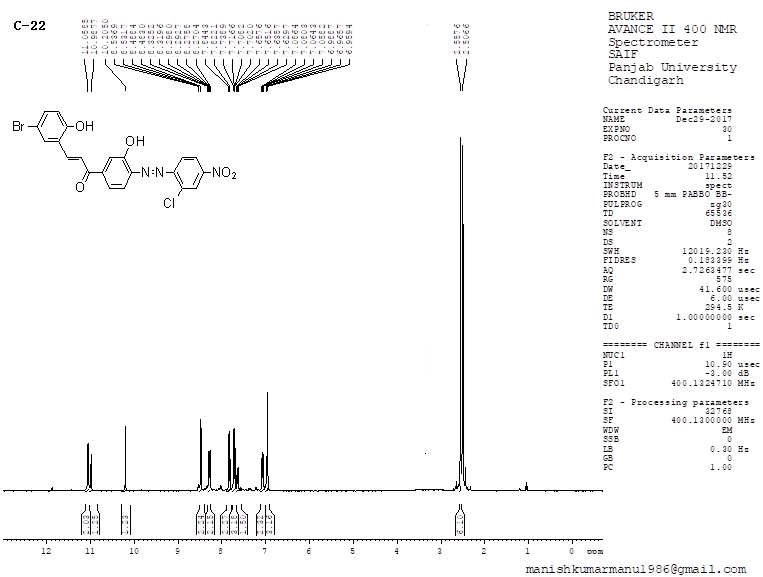

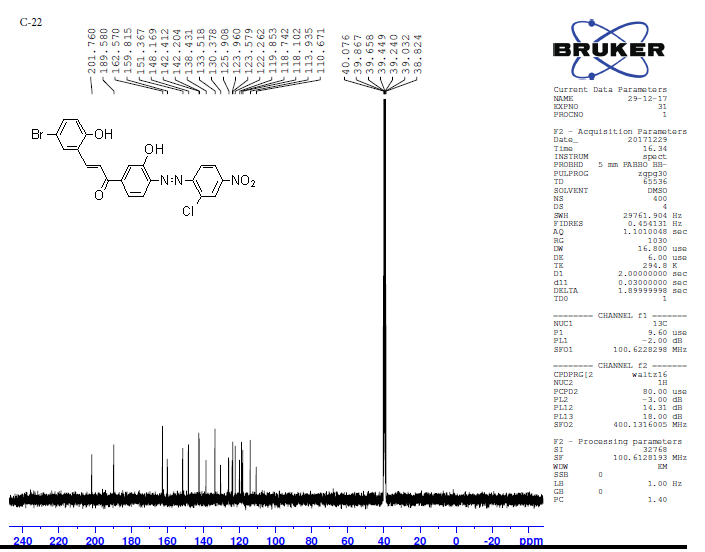

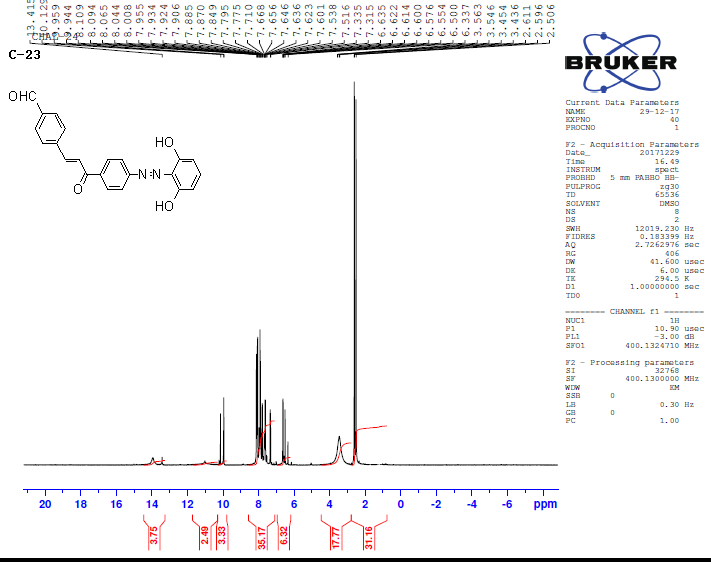

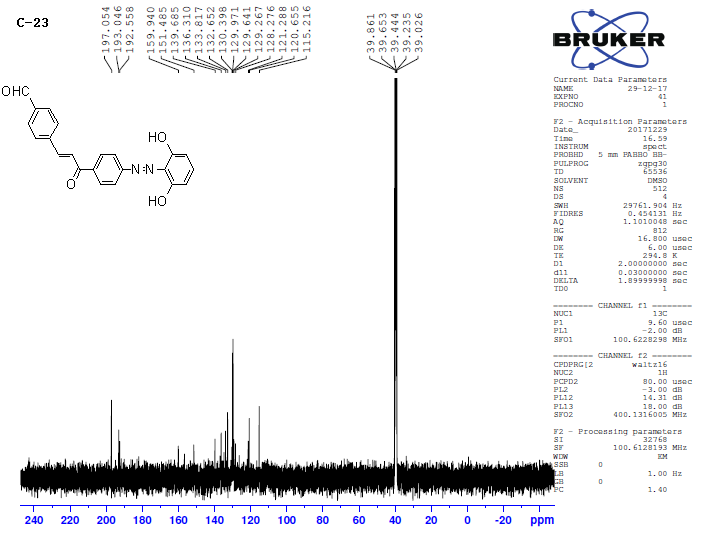

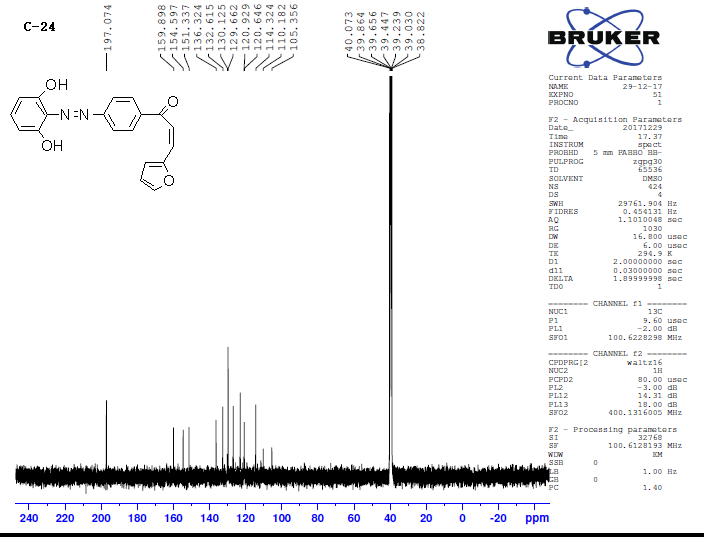

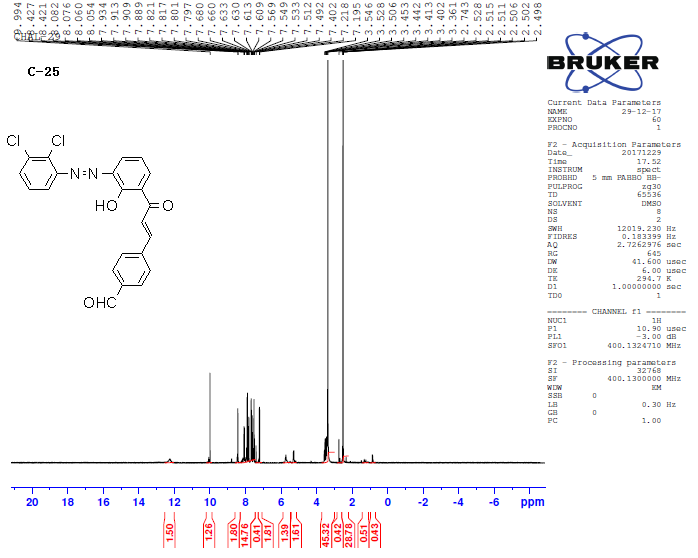

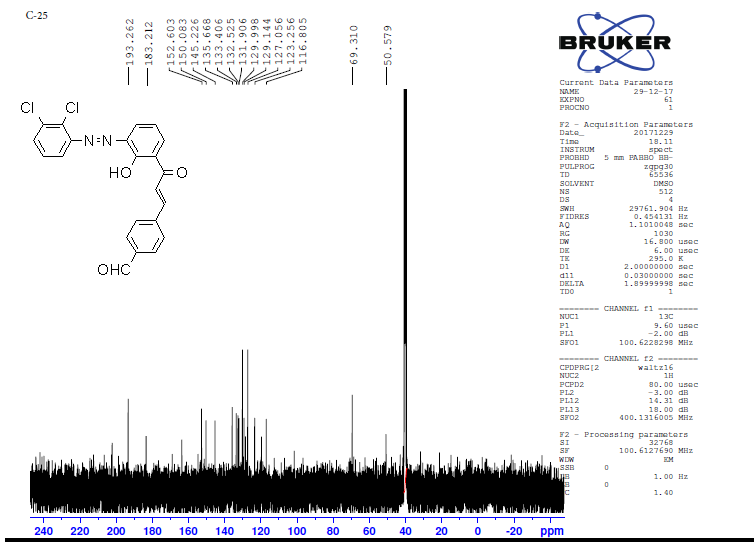

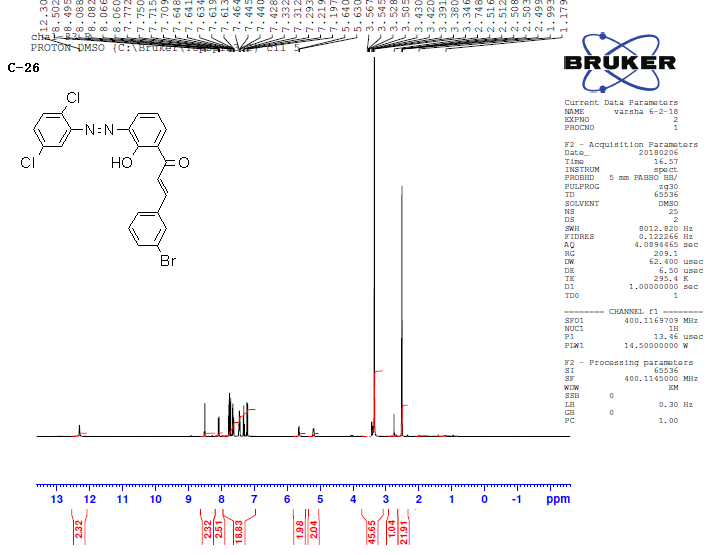

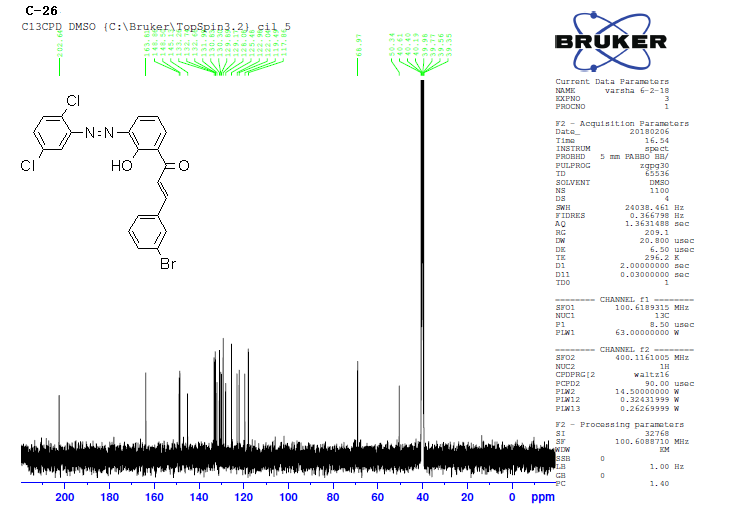

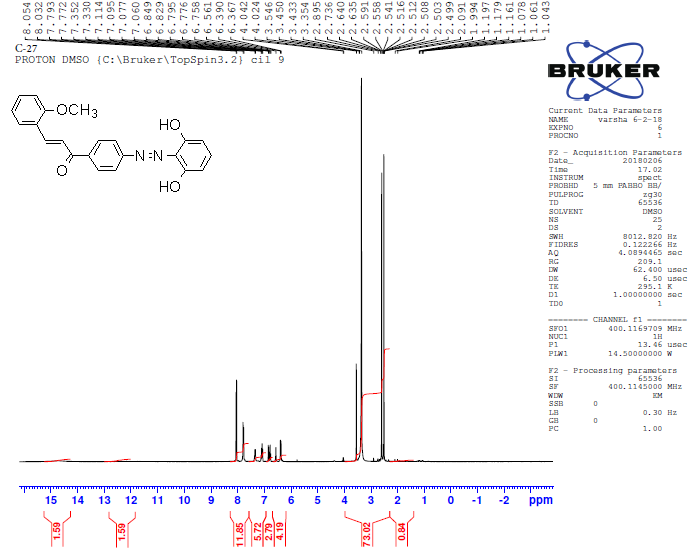


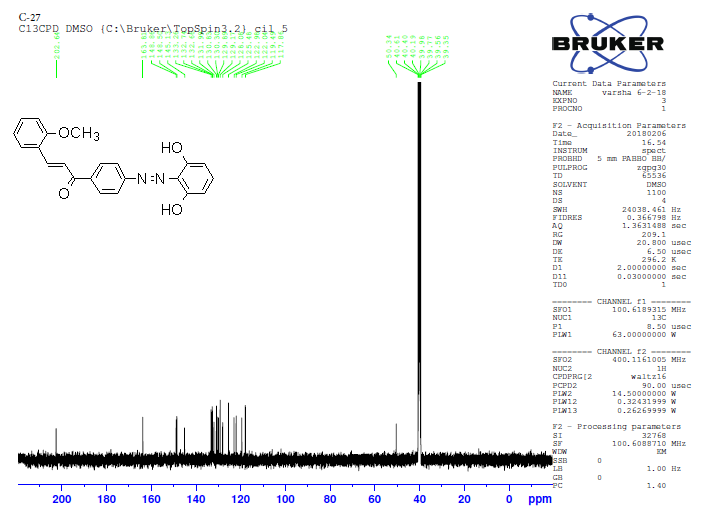

Supplement: Supplementary file 1 — Additional file 1. 1H and 13C NMR data of most active compounds has been provided. [file 13065_2019_596_MOESM1_ESM.docx]
